# Computational modeling of methionine cycle-based metabolism and DNA methylation and the implications for anti-cancer drug response prediction

**DOI:** 10.18632/oncotarget.24547

**Published:** 2018-02-21

**Authors:** Mengying Zhang, Christian Saad, Lien Le, Kathrin Halfter, Bernhard Bauer, Ulrich R. Mansmann, Jian Li

**Affiliations:** ^1^ Institute for Medical Informatics, Biometry and Epidemiology, Ludwig-Maximilians University of München, Munich, Germany; ^2^ German Cancer Consortium (DKTK), Heidelberg, Germany; ^3^ German Cancer Research Center (DKFZ), Heidelberg, Germany; ^4^ Department of Computational Science, University of Augsburg, Augsburg, Germany

**Keywords:** metabolism, methylation, chemotherapy, molecular modelling, treatment prediction

## Abstract

The relationship between metabolism and methylation is considered to be an important aspect of cancer development and drug efficacy. However, it remains poorly defined how to apply this aspect to improve preclinical disease characterization and clinical treatment outcome. Using available molecular information from Kyoto Encyclopedia of Genes and Genomes (KEGG) and literature, we constructed a large-scale knowledge-based metabolic *in silico* model. For the purpose of model validation, we applied data from the Cancer Cell Line Encyclopedia (CCLE) to investigate computationally the impact of metabolism on chemotherapy efficacy. In our model, different metabolic components such as MAT2A, ATP6V0E1, NNMT involved in methionine cycle correlate with biologically measured chemotherapy outcome (IC50) that are in agreement with findings of independent studies. These proteins are potentially also involved in cellular methylation processes. In addition, several components such as 3,4-dihydoxymandelate, PAPSS2, UPP1 from metabolic pathways involved in the production of purine and pyrimidine correlate with IC50. This study clearly demonstrates that complex computational approaches can reflect findings of biological experiments. This demonstrates their high potential to grasp complex issues within systems medicine such as response prediction, biomarker identification using available data resources.

## INTRODUCTION

Over the past decade, various studies have discovered a number of metabolic changes that promote or support cancer development [1, 2]. Cancer metabolism has been considered a hallmark of cancer, which has been linked to the initiation, metastasis, and recurrence of cancer [3–5]. Therefore, understanding the pathways and mechanisms of cancer metabolism holds promise for improving patient drug treatment [6, 7]. In this regard, several recent studies provide evidence that serine metabolism is an essential energy source for cancer development, which make this serine-based metabolic pathway a potentially druggable target [8, 9]. In parallel, a number of studies in the past decade have investigated the complex role of epigenetics in human cancer [10–12]. Epigenetic regulations such as DNA methylation, histone modification, and nucleosome remodeling can influence diverse biological processes that are fundamental to the initiation and development of cancer [13].

As the fields of cancer metabolism and cancer epigenetics have developed, so has the appreciation of the functional crosstalk between these processes [14–16]. Recent studies provide strong evidence that changes in metabolism of cancer cells can directly or indirectly impact epigenetic regulation, which leads to the promotion of cancer development [17–19]. Clear evidence has shown that metabolic alteration affecting protein and DNA methylation are a potential driving force for cancer development. For instance, it has been shown that the metabolic enzyme nicotinamide N-methyltransferase (NNMT) is overexpressed in a variety of human cancers. The high expression of this enzyme enhances cancer aggressiveness by broadly changing methylation profiles [20, 21]. Moreover, Shyh-Chang et al. reported that embryonic stem cells strongly depend on threonine to maintain S-adenosyl methionine (SAM) synthesis, an essential primary methyl donor. The condition of threonine starvation leads to a dramatic decrease of histone methylation with a subsequent strong inhibition of proliferative activity [22]. The cyclic conversion of different methionine-based derivatives such as SAM with help of enzymes including NNMT, is referred to as the methionine cycle. This cycle provides methyl units for a variety of methylations for proteins, DNA, RNA, lipids and others. Some other studies have shown that the purine synthesis pathway is highly upregulated in cancer. This pathway is not only responsible for its contribution to the synthesis of nucleic acids (RNA and DNA), but also for the production of large amounts of ATP to meet the high energy demand characteristic for cancer development [23, 24]. All these facts provide evidence to the fact that altered metabolic pathways with relation to cancerous methylation may prove fundamental in cancer development or drug treatment.

In a recent review of Mc Auley et al. on a series of computational models with focus on folate metabolism and methylation, the authors state that a model of this kind might be able to provide an ideal framework for handling the complexities of physiologic and pathologic states [25]. In another study an integrative model of DNA methylation was constructed by integrating multi-platform data from thousands of human tumors to explain the important relationship between metabolism, methylation, and their respective clinical implications [26]. However, this study only focused on statistical approaches, neglecting molecular and cellular regulation mechanism such as transcriptional and translational regulations.

In our previous study we constructed a genome-scale *molecular signaling model* (MSM) containing multiple cancer-relevant signaling pathways and different cancer hallmarks [27]. Using an *in silico* approach and integrating molecular models with genetic information such as gene expression data we were able to effectively handle complex issues such as prediction of targeted treatment outcome. Moreover, it was also shown that the costs of an *in silico* approach both in regard to time and materials is much lower compared to conventional *in vitro* based studies such as cell line, xenograft and other experimental settings [27–29]. However, the MSM did not consider any aspects of metabolism and is therefore not able to fully reflect the metabolic regulation of carcinogenesis. As stated previously, diverse metabolic mechanisms might be key factors to investigate and predict the therapeutic effect of targeted or broadly acting cancer treatment. Therefore, we investigated whether a comprehensive modeling of metabolism with focus on epigenetic regulation might be able to clarify the intricate relationship between cancer treatment and cancer metabolism. Furthermore, it is unclear how the relationship between cancer metabolism and methylation can be used for individualized treatment outcome predication. Nevertheless, the application of large-scale metabolic models which reflect the metabolic behavior of cancer cells hold great promise for a more refined, systems approach in clinical cancer treatment [30]. The aim of our study was the application of a molecular modeling procedure in order to construct a large-scale metabolic model and its pre-clinical validation regarding treatment prediction. We intend to use this model to investigate the methionine cycle-based molecular metabolic function and to compare it with experimental key findings in this field.

## RESULTS

### Study design and construction of the methionine cycle-based metabolic model (MCPM)

**Figure 1:**
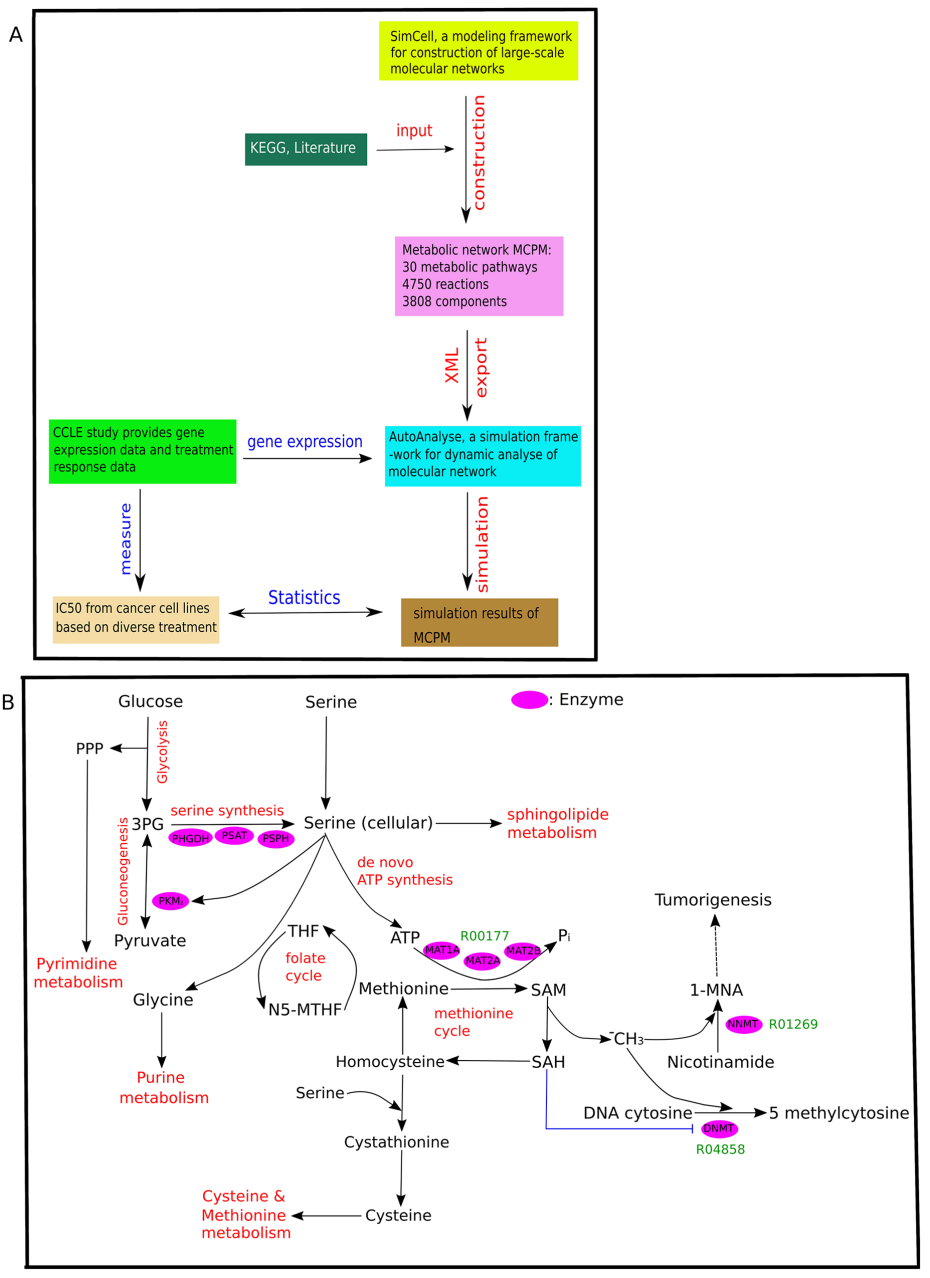
Overview of MCPM **(A)** The flowchart of the applied methods. The metabolic network MCPM was constructed by SimConCell and based on KEGG database and current literature. Then MCPM is exported as an XML file, which is an input file for simulation in AutoAnalyse to simulate a molecular model with gene expression data. Lastly, spearman analysis was used to investigate the correlation between simulation value of components in the model and drug treatment (IC50 value) from 30 types of cancer cell lines (CCLE). **(B)** The schematic shows MCPM and its crosstalk with other metabolic pathways. The Key enzymes are shown in pink. Key reactions are shown in green. R00177 (Orthophosphate + Diphosphate + S-Adenosyl-L-methionine <=> ATP + L-Methionine + H2O), R04858 (S-Adenosyl-L-methionine + DNA cytosine <=> S-Adenosyl-L-homocysteine + DNA 5-methylcytosine), R01269 (S-Adenosyl-L-methionine + Nicotinamide <=> S-Adenosyl-L-homocysteine + 1-Methylnicotinamide).

Figure 1A summarizes the basic workflow: The study selectively used molecular information obtained from publicly available research databases and literature to construct a large-scale molecular metabolic network (MCPM). After model construction, gene expression data from different cancer cell lines was integrated for *in silico* simulation. The simulation results for protein components of the model were subsequently used to calculate correlations with the IC50 of different drug treatment from various cancer cell lines taken from the treatment data of Cancer Cell Line Encyclopedia (CCLE) [31]. We focused on broad-acting chemotherapy treatment, specifically DNA-Topoisomerase (irinotecan and topotecan from CCLE) and Histone-Deacetylase (HDAC) inhibitors (panobinostat). It is of interest to study how the MCPM reflects the mode of action determined through different properties of cancer metabolism [3, 19, 32–34].

The model construction of this study is mainly based on data obtained from the KEGG data base (http://www.genome.jp/kegg/) [35] and literature research. The constructed MCPM consists of 30 pathways, 4750 reactions, and 3755 components involving gene, mRNA, protein, compound and pseudo-object (Table 1). The transcription and translation reactions determine the relationship between gene, mRNA, and protein. Its central part is the methionine cycle pathway (MCP) and its direct crosstalk with other metabolic pathways including glycine, serine, threonine, cysteine, methionine, purine, and pyrimidine metabolism, as well as glycolysis and gluconeogenesis (Figure 1B). Therefore, we refer to this model as the MCPM (methionine cycle pathway-based model). Input starts with 3-phosphoglycerate (3PG), a precursor for serine synthesis and activator of AMP-activated protein kinase (Figure 1B). The 3PG are successively catalyzed by the enzymes PHGDH, phosphoserine aminotransferase (PSAT), and phosphoserine phosphatase (PSPH). PSAT converts glutamate and alpha-ketoglutarate to support the synthesis of serine, which in turn supports one-carbon metabolism for downstream conversion within the methionine and folate cycle. The folate cycle recycles methionine that has been synthesized from homocysteine. In the model, MAT1A and MAT2A/B catalyzes the reaction from methionine to SAM. Nicotinamide N-methyl-transferase (NNMT) catalyzed the conversation of Nicotinamide to 1-methylnicotinamide (1-MNA) by using SAM as a methyl donor. Serine supports SAM synthesis through methionine and de novo ATP synthesis. The model ends with a DNA methylation process catalyzed by DNA methyltransferase (DNMT) enzyme, which is dependent on the amount of donor methyl obtained from SAM.

**Table 1:** Component and reaction summary of the model MCPM

Component	No.	Reaction	No.	Pathway	No.
Gene	786	Transcription	790		
mRNA	1582	Translation	779		
Protein	794	Decay	1571		
Metabolite	582	Translocation	791		
pseudo-Object	11	Metabolism	819		
Sum:	3755	Sum:	4750	Sum:	30

### Simulation and analysis of CCLE Data

**Figure 2:**
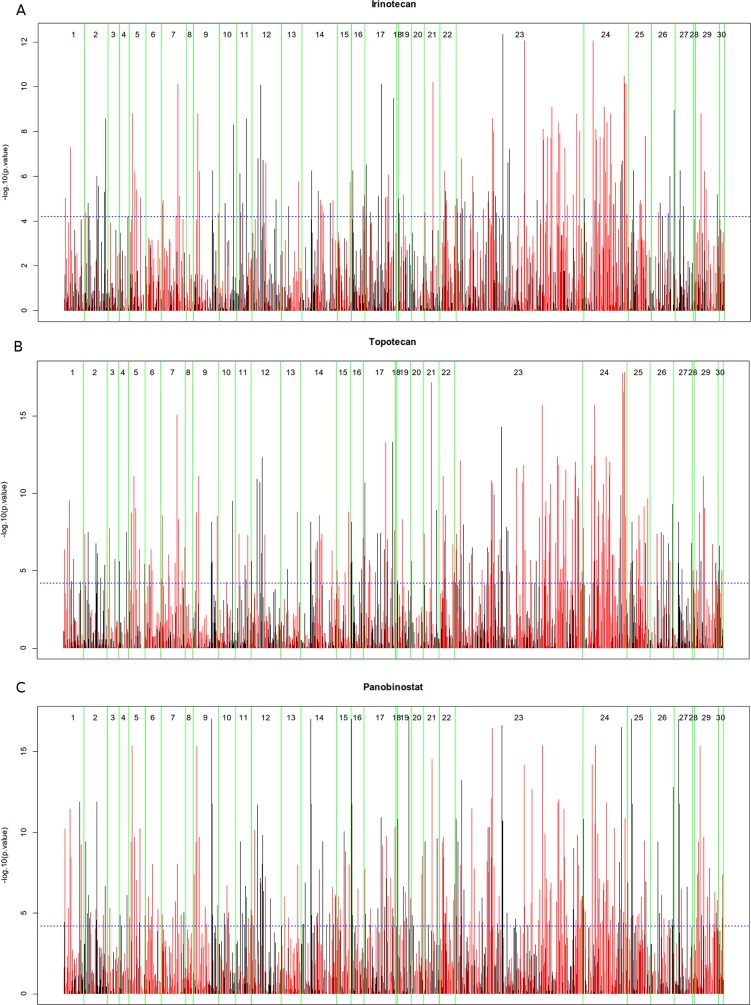
Manhattan Plots of drug treatment (IC50) with simulation of MCPM consisting of 30 metabolic pathways **(A)** irinotecan, **(B)** topotecan, **(C)** panobinostat. If the bar is red, the correlation is negative (high model based values have a low IC50, high values related to good response), if the bar is black, the correlation is positive (high model based values have a high IC50, high values related to bad response).

We utilized the gene expression data of Cancer Cell Line Encyclopedia (CCLE) [31] and incorporate these data with MCPM into AutoAnalysis (Materials and Methods). Afterwards, we performed a graphic-based data-flow analysis simulation in order to investigate whether this type of metabolic simulation could reveal the inhibition effect of different chemotherapies within the CCLE. We investigated response data of all chemotherapy drugs from CCLE: irinotecan, topotecan, panobinostat, paclitaxel and 17-AAG. Response data (IC50) of the cell lines under these drugs were correlated with simulation values of components from the model MCPM. The results of the correlation analysis regarding MCPM protein components and IC50 are presented for each drug in a specific Manhattan-like plot which is separated into the strips of 30 pathways and bars whose length represents the p-value of the corresponding correlation (Figure 2).

These five plots are given in the Figure 2 and Supplementary Information 1. Lists with pathway specific correlation results of the protein components are also given in the Supplementary Information 1. The results show that overall correlations between the simulation values of model components and response data from each of irinotecan, topotecan and panobinostat are significant, whereas the correlations from paclitaxel, 17-AAG are not significant. Therefore, this following study mainly focused on irinotecan, topotecan and panobinostat.

### Correlation of model components and DNA-topoisomerase inhibitor efficacy

**Figure 3:**
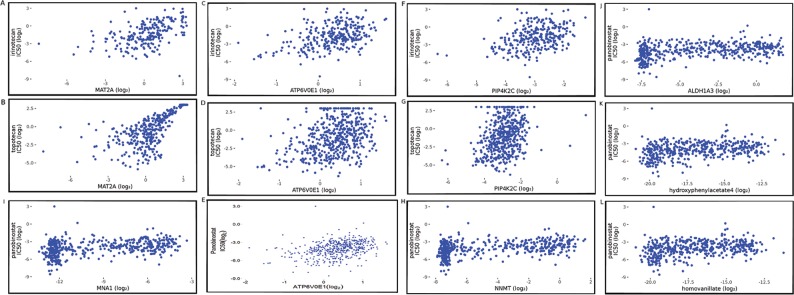
Spearman correlation plots between simulation value of MCPM components and IC50 value of drug treatments **(A)** MAT2A vs. irinotecan (spearman: 0.5293110 p=1.250274e-16); **(B)** MAT2A vs. topotecan (spearman: 0.6808339 p=7.988427e-14); **(C)** ATP6V0E1 vs. irinotecan (spearman: 0.4056839 p=4.483729e-13); **(D)** ATP6V0E1 vs. topotecan (spearman: 0.3491871 p=4.875323e-15); **(E)** ATP6V0E1 vs. panobinostat (spearman: 0.3775625 p=2.262852e-17); **(F)** PIP4K2C vs. irinotecan (spearman: 0.3562168 P=3.188388e-10); **(G)** PIP4K2C vs. topotecan (spearman: 0.3369385 p=4.778644e-14); **(H)** NNMT vs. panobinostat (spearman: 0.3829126 p=7.334179e-18); **(I)** 1-MNA (MNA1) vs. panobinostat (spearman: 0.3723424 p=4.231325e-14); **(J)** ALDH1A3 vs. panobinostat (spearman: 0.3818062 p=9.274430e-18); **(K)** 4-Hydroxypheinylacetate vs. panobinostat (spearman: 0.3323161, p=1.3982196e-13) **(L)** homovanillate vs. panobinostat (spearman: 0.3288698, 2.569935e-13).

The mechanism of chemotherapy based on DNA-topoisomerase inhibition acts via inactivation of the enzymatic function of DNA-topoisomerase to prevent DNA replication and transcription processes in highly prolific cells. This irreversibly leads to apoptosis [32, 36]. In the MCPM, we found that a simulation value of the enzyme protein MAT2A correlates with the IC50 value of irinotecan (spearman: 0.5293110 p=1.250274e-16) and topotecan (spearman: 0.6808339 p=7.988427e-14) (Figure 3A-3B). This result shows that the high MAT2A simulation value is associated with a lower response (high IC50 value) to DNA-topoisomerase inhibitors. A high MAT2A simulation value can generate more input for the methionine-cycle, which increases the activity and output of the methioninie-cycle during simulated metabolism. This cycle mainly provides methyl units for methylation reactions catalyzed by DNMT, which results in hypermethylation, especially DNA methylation [14, 37]. This correlation between MAT2A and treatment efficacy of irinotecan and topotecan indicates that high levels of DNA methylation may help cells become more resistant against DNA-topoisomerase inhibition. This finding is in agreement with other important findings that suggest that DNA hypermethylation can be considered a potential chemotherapy target [19]. In addition, several studies have shown that cancer cells have a high demand for serine that can play a key role in feeding one-carbon units to supports both nucleotide synthesis and NADPH production [38–41]. During model simulation with gene expression data from the CCLE, simulation values of serine from all cell lines are critically low, even with a 10-folder increase of serine input. These results indicate that MCPM requires a large amount of serine during simulated metabolism, which was also seen in other studies [38–41].

The simulation value of ATP6V0E1 in the MCPM also correlated with the IC50 value of irinotecan (spearman: 0.4056839 p=4.483729e-13) and topotecan (spearman: 0.3491871 P=4.875323e-15) in the CCLE (Figure 3C-3D). This protein is responsible for the cellular ion transport and plays a role in the activation of the immune system [42]. The significant correlation between ATP6V0E1 and IC50 values suggests that the activation of the immune system induced by ATP6V0E1 may counteract the inhibitory effect of DNA-topoisomerase. However, future studies need to confirm this finding in an *in vivo* experimental setting.

The simulation results showed that the simulation value of the enzyme PIP4K2C in MCPM correlated with the IC50 value of irinotecan (spearman: 0.3562168 P=3.188388e-10) and topotecan (spearman: 0.3369385 p=4.778644e-14) (Figure 3F-3G). The PIP4K2C protein is mainly involved in the pathway of inositol phosphate metabolism. This metabolic pathway has tight crosstalk with diverse signaling pathways such as PI3K-AKT, MAPK, and AMPK among others [43]. The upregulation of PIP4K2C could strengthen the crosstalk between these important pathways, thereby counteracting the inhibition of DNA-Topoisomerase. Moreover, our results show that the simulation value of several components from the purine and pyrimidine pathways are significantly correlated with the IC50 value of irinotecan and topotecan (Table 2). This indicates that a high amount of purines and pyrimidines within cancerous cells might protect against DNA-topoisomerase inhibition.

**Table 2:** A list of components that present high spearman correlation in the MCPM regarding irinotecan and topotecan treatment

Drug type	Component	Metabolic pathway	Correlation / P value
Irinotecan	ATP6V0E1	Purine	0.4056839 p=4.483729e-13
Irinotecan	ADCY6	Purine	0.3115568 p=5.745343e-08
Topotecan	ATP6V0E1	Purine	0.3491871 p=4.875323e-15
Topotecan	PIP4K2C	Purine	0.3562168 p=3.188388e-10
Topotecan	CMP	Pyrimidine	0.3231642 p=2.184534e-10
Topotecan	ATP6AP1	Purine	0.2899188 p=1.242801e-10

These components and reactions are from purine and pyrimidine pathways

### Correlation between model components and inhibition effect of histone-deacetylase (HDAC)

Histone proteins are responsible for chromatin configurations, which may function as a control switch between gene transcription and gene silence [44]. Therefore, the cellular activity of transcriptional factors, tumor suppressors, structural proteins, and other important cellular regulators are highly dependent on the stages of chromatin configurations. This places HDAC in a unique position to affect a myriad of cellular processes including proliferation, apoptosis, and metastasis. We studied the treatment effect (IC50 value) of panobinostat, a Histone-Deacetylase inhibitor in the CCLE and analyzed the association of these values and the MCPM simulation values. Our results show that simulation values of NNMT and 1-MNA correlate with the IC50 value of panobinostat (spearman: 0.3829126 and 0.3723424; p<0.05) (Figure 3H-3I). NNMT is a special metabolic enzyme, which exerts specific control over cells methylation potential thereby broadly impacting the epigenetic state of cancer cells [21]. Diverse studies have demonstrated that 1-MNA has pro-angiogenic activity, anti-thrombotic activity, anti-inflammatory activity, and vasoprotective properties influencing cancer metastasis [45–48]. Our result shows that the high simulation value of NNMT and 1-MNA correlates with low treatment response of CCLE regarding panobinostat treatment, thereby potentially signaling that an effect of histone modification on gene expression regulation is tightly related to the cellular methylation state. However, more studies are necessary to clarify the association of HDAC inhibition with clinical findings.

Regarding HDAC inhibition our results also found that the simulation value of ALDH1A3 correlates to the IC50 of panobinostat (spearman: 0.3818062 9.274430e-18) (Figure 3J). ALDH1A3 has been shown to play an essential role in treatment resistance to chemotherapy and radiotherapy [49–51]. This protein is deeply involved in the regulation of diverse signaling and metabolic pathways and is therefore considered a potential biomarker for cancer stem cell (CSC) [52–54]. The hypermethylation of the ALDH1A3 gene promoter has previously been reported in various tumors [55, 56]. This correlation between ALDH1A3 and the panobinostat IC50 value might be an indication that the mechanism of ALDH1A3 for resistance of chemotherapy is based on the relationship between the histone-modification of gene expression and methylation. This link may generally protect cells from dysfunctions in the DNA replication process. The simulation value of ATP6V0E1 a regulator of immunsystem, also correlated with the IC50 of panobinostat (spearman: 0.3775625 2.262852e-17) (Figure 3E). This indicates that ATP6V0E1 might be a potential prognostic biomarker to predict HDAC inhibition.

The simulation value of 4-Hydroxyphenylacetate and homovanillate correlated with the IC50 of panobinostat (spearman: 0.3323161 and 0.3288698; p<0.05) (Figure 3K-3L). Both metabolic components have been validated as sensitive metabolic biomarkers in cancer screening [57]. Both components are key components in the tyrosine metabolic pathway where the enzyme ALDH1A3 plays an essential role in the methylation processes [3]. In addition, simulation values of several components such as 3, 4-dihydoxymandelate, PAPSS2, UPP1, Uracil, NT5E correlated with the IC50 of panobinostat (Table 3). These components are part of the purine and pyrimidine metabolism pathways, both of which are strongly upregulated in cancer cells and provide nucleic acids necessary for cellular proliferation and tumor growth [23, 24].

**Table 3:** A list of components that present high spearman correlation in the MCPM regarding panobinostat

Drug type	Component	Metabolic pathway	Correlation / P value
Panobinostat	ATP6V0E1	Purine	0.3775625 p=2.262852e-17
Panobinostat	UPP1	Pyrimidine	0.3762676 p=2.963006e-17
Panobinostat	3,4-Dihydroxymandelate	Tyrosine	0.3723126 p=3.021123e-17
Panobinostat	PAPSS2	Purine	0.3375621 p=5.454525e-14
Panobinostat	NT5E	Purine, pyrimidine	0.3049326 p=1.431208e-11

These components and reactions are from purine, pyrimidine and tyrosine pathways

### Computational aspects

In our study *in silico* simulation was performed for all of the 479 cancer cell lines that were part of the CCLE in order to investigate the metabolic behavior of these cell lines. The entire simulation procedure lasted 16 min and 43 seconds. The simulation was conducted on one laptop with a hardware that consisted of 2 cores, 2GB RAM, and 8GB memory. The subsequent spearman correlation was repeated a total of 7.071 million times between the IC50 of each drug treatment and simulation value of model components. This statistical analysis ran for a total of 19 min and four seconds to summarize and achieve the above shown results.

## DISCUSSION

Recent evidence has shown that alterations in metabolism that affect cellular methylation may be a significant driving force in carcinogenesis. This means that cancer cells are able to take advantage of this relationship to influence gene expression, chromatin structure, and cellular function thereby enabling treatment resistance and cellular proliferation [17, 20]. Given these facts, hypermethylation has been suggested as a potential drug target [19]. In order to explore this intricate relationship between metabolism, methylation, and cytostatic drug efficacy we constructed the molecular model MCPM based on methionine cycle-based metabolism and related metabolic pathways. We performed an *in silico* simulation with the MCPM using the AutoAnalysis integrated with gene expression data from the CCLE, containing more than 479 cancer cell lines from approximately 30 types of cancer. We furthermore utilized the drug treatment data (IC50 value) of the CCLE and compared this response data to the simulation data of the MCPM.

The results show that distinct components from the MCPM play important roles as biomarkers for treatment efficacy and prognosis, specifically regarding the inhibition of DNA-topoisomerase and HDAC. These components are namely MAT2A, NNMT, ATP6V0E1, PIP4K2C, ALDH1A3, 4-hydroxyphenylacetate, and homovanillate among others. Many of these results are in agreement with findings from other independent studies, which indicate a successful model validation. In detail, during simulation within the model MCPM, the protein MAT2A can push the methionine cycle to generate more SAM. When the key metabolic enzyme NNMT is available, then 1-MNA would be generated from SAM and nicotinamide. 1-MNA can enhance activity of the cyclooxygenase 2 (COX-2) pathway and increase angiogenesis to protect cancer cells from the inhibition of DNA-topoisomerase through chemotherapy [33]. Our results reflect those found in a study by Mehrmohamadi et al (2016). Their results also show that MAT2B together with the methionine cycle has a potential to be predictive [26]. Diverse studies provide evidence that NNMT is a putative onco-metabolic protein, which can promote tumorigenesis by widely changing cellular methylation pattern via control over the availability of free methyl units. This enzyme protein is also overexpressed in different types of cancer [58–62]. The simulation value of NNMT and 1-MNA were also predictive for the efficacy of HDAC inhibition.

The common basis for both types of drug inhibition pathways is the methionine cycle. It controls the methylation resources within cells (including cancer cells). A strong upregulation of this cycle leads to abundant amounts of methyl units, thereby providing a necessary precondition for hypermethylation. This state of hypermethylation might substantially protect cancer cells from the therapeutic effect of these chemotherapy drugs.

Furthermore, the results show that the simulation value of ATP6V0E1 correlates with the IC50 of irinotecan, topotecan, and panobinostat. ATP6V0E1 belongs to V-ATPase family, whose members are general highly expressed in cancer cells to control the acidity of microenvironment so that metastasis and the epithelial-mesenchymal transition are promoted [63]. Our simulation result indicates that the higher the simulation value of ATP6V0E1 is the lower the treatment response. This is confirmed by findings that cancer cells with high concentration of ATP6V0E1 or other V-ATPase family members are more resistant to anti-neoplastic drugs [34, 64, 65]. Therefore, based on this simulation result, we would propose ATP6V0E1 as a potential predictive biomarker for DNA-topoisomerase and HDAC inhibition based on *in silico* simulation of treatment outcome. Future studies will be focusing on expanding the MCPM to incorporate other V-ATPase family members.

Our results also show that the simulation value of several components from the purine and pyrimidine pathways in the MCPM were significantly associated with the efficacy of these two types of cytostatic drugs. This finding indicates that cancer cells need even larger amounts of nucleic acids for survival and proliferation to cope with such a therapeutic intervention. The simulation results indicate that the more nucleic acids cancer cells receive during the course of drug cancer therapy, the more resistant the cancer cells would become. Moreover, we noticed that the serine demand is always high during the simulation of CCLE for the included 30 types of cancer. The simulation of MCPM shows that no free methyl units remain when the serine resource is artificially cut off, leading to a general state of hypomethylation. This scenario might represent an effective way to induce detrimental effects on cancer cells as suggested by other studies [40, 41, 66]. Nevertheless, a limitation of the MCPM is that this large-scale model was constructed with a focus on the methionine cycle and its related metabolic pathways. That makes approximately 40% of knowledge originating from KEGG. The crosstalk between signaling and metabolic pathways, as well as the microRNA regulation effect on the metabolism were also not been considered during this study. Moreover, the model does not take some biological reaction types such as post-translation modification and protein-protein interactions into consideration. Future studies will be focused on improving the model according to these limitations.

In our study, we were able to demonstrate the high efficiency of a computational systems approach. Our *in silico* simulation ran 34 minutes and 47 seconds in total, the same amount of work would normally require 200 working days with four co-workers in a wet-lab. Moreover, recent studies have demonstrated that treatment with triple-drug-combinations appear to be most effective in comparison to single drug treatment [67, 68]. Verification of these multi-drug combination treatments are hard to replicate in the setting of a wet-lab, here computational simulation could be a clear alternative. This line of research will be the next step of our research objective.

After this model validation study, our next steps of our research will be based on the strong correlations found in the CCLE data between computational components of our model and the biological treatment effect quantified by the IC50. The strong correlation motivate us to use linear penalized regression which allow to model prediction scores for treatment efficacy regarding irinotecan, topotecan, and panobiostat. We will evaluate these prediction scores with regard to treatment response on patients from the TCGA which were treated with one of the substances of interest.

Based on our experience in previous studies [27–29], we think that many studies of gene signature are premature in analyzing the pre-clinical and clinical outcome. While big data such as gene expression contains a multitude of valuable information, intricate biological regulation mechanisms such as transcriptional and translational regulation, feedback-control regulation, ligand-competitor mechanism and others are not included within any kind of big data. Biological modeling could become an essential step to fill the gap for a meaningful application of big data.

## MATERIALS AND METHODS

### Study design

**Figure 4:**
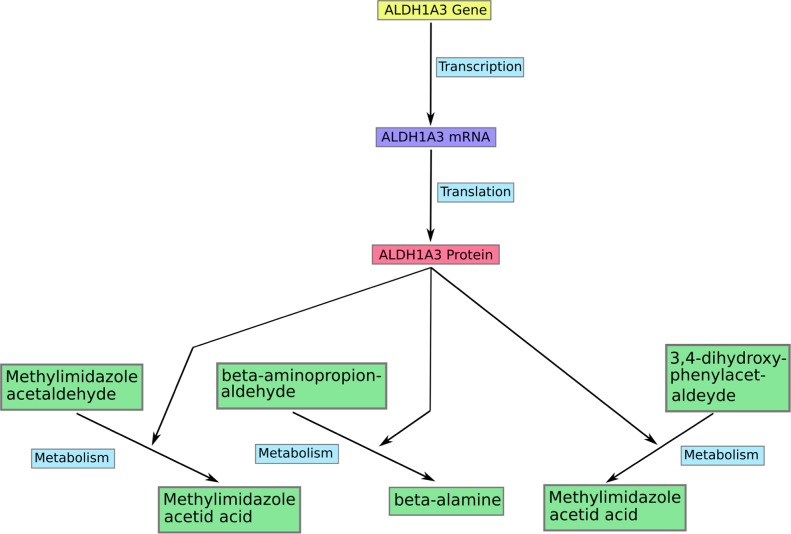
An example of modeling in the model MCPM Each gene from the model participate in a transcription reaction to produce a mRNA. And a mRNA takes part in translation reaction to produce a corresponding protein in the model. The protein acts in most cases as enzyme to catalyse different metabolic reactions such as ALDH1A3 in the figure.

This study selectively used the molecular information of metabolism from KEGG (http://www.genome.jp/kegg/) to construct a large scale metabolic network containing 627 metabolites, 786 genes, 794 proteins, and 30 metabolic pathways such as (glycolysis, pentose_phosphart pathways among others). The unpublished web-based modeling software SimConCell was used to compile the KEGG information (structure and components of pathways, quantitative information on reactions within the pathway) into a formal network structure which is the structural basis for the MCPM model construction. SimConCell formalizes each model component (including gene, RNA, protein, metabolite, and other) as a node and each biochemical reaction as an edge to link corresponding nodes (Figure 4). The output of the SimConCell software is an XML file available under Supplementary Information 4. The gene expression data of different cancer cell lines are integrated into the model during *in silico* simulation. The simulation procedure has been implemented using the AutoAnalyse framework developed by the informatics department of the Augsburg University.

### SimConCell for molecular modeling

SimConCell is designed as web-based systems biology software for development, simulation, and analyse of molecular models for cellular reaction networks. It provides a number of functions to support users to design and construct larger-scale molecular models such as semi-automation option for definition of transcription and translation reactions. Furthermore, SimConCell functions as a model repository and can split or merge different models, thereby enabling specific model construction and analysis. Each entity in a SimConCell model including gene, RNA, protein, compound, complex among others can be associated with publicly available IDs such as Ensemble-ID, UniProt, ChEBI-ID. Given these putative IDs, models from SimConCell can be easily integrated with different genetic data including gene expression data, protein data, and metabolic data.

### Literature search

The literature search was conducted using available publications on Google scholar and PubMed. We used ‘cancer metabolism’, ‘targeting cancer therapy’ as key words. The search yielded 1,440,000 and 1,430,000 sources respectively. Using the advanced search key words were applied, such as ‘metabolic target’ AND ‘cancer therapy’ (A), ‘targeting metabolism’ AND ‘cancer therapy’ (B), ‘metabolic transformation’ AND ‘cancer’ (C). Results are limited by review, abstract availability, and publication within the past ten years (2006-2017). Our final search results were 542 (A), 5011 (B) and 416 (C) results. We studied the top ranking 100 publications that are associated with relevant content on epigenetics und cancer metabolism.

### Gene expression data and drug response data of cancer cell lines

The study of Barretina and colleagues has established the Cancer Cell Line Encyclopedia (CCLE) [31] through systematically analysis of drug responses of 479 cancer cell lines derived from 30 solid and hematological cancer types, which allows identification of genetic, lineage, and gene-expression-based predictors of drug sensitivity. These gene expression data of CCLE were generated for each of these cell lines using Affymetrix U133 plus 2.0 array. The data was pre-treatment data and not normalized. It is associated with accession number GSE36139 from Gene Expression Omnibus (GEO). The drug response data of CCLE were generated as pharmacological sensitivity *in vitro* and can be accessed via http://www.broadinstitute.org/ccle.

### Simulation procedure of autoanalyse

The AutoAnalyse framework supports a data-flow-based network simulation with the central point of graphic manipulation (a detailed description of the software is currently under submission). Different types of components from our metabolic model will be translated into the defined instance objects within a model-based representation of AutoAnalyse. The input model file for AutoAnalysis is the XML file of the model Supplementary Information 4. The gene expression data of CCLE is also translated into a XML file for AutoAnalysis (Supplementary Information 2) (Figure 1A).

For instance of a reaction translation: the input concentration for a reaction ∈ R and role ∈ {e, g, i, s, tr(a), tr(r)} (e: enzyme; g: gene; i: inhibitor; s: substrate; tr(a): transcriptional activator; tr(r): transcriptional repressor) is the product of all reactant object concentrations which belong to the reactant of the respective role:

$$I(\text{reaction},\text{role})=\underset{\text{object}.\text{concentration}}{\Pi }\text{ object}\in \text{reactant }(\text{reaction},\text{role}).\text{objects}$$

The output concentration for reaction ∈ R is computed by applying the kinetic rate law to the required and optional input concentrations of the reaction. If the object C is the product of a reaction with substrates of the object A and object B, and the C does not participate in any other reaction in the model, then the concentration of [C] = reaction kinetic law ([A], [B]). All kinetic rate laws applied in the AutoAnalysis are listed in the Supplementary Information 3.

### Statistical analysis

We analyze the correlation (using Spearman correlation coefficient) between the numerical values of a “protein” model component with the biologically measured IC50. The results of the analysis are presented for each of the 5 substances under study in a Manhattan-like plot which shows (per pathway) the transformed p-value (-1)·log_10_ (p-value) of the corresponding correlation analysis. We apply Bonferroni-adjustment for multiple testing per substance.

## SUPPLEMENTARY MATERIALS










