# Supplementary material for: Computational modeling of methionine cycle-based metabolism and DNA methylation and the implications for anti-cancer drug response prediction
Source: Oncotarget. 2018 Feb 21;9(32):22546–58. doi: 10.18632/oncotarget.24547 (PMC5989406; doi:10.18632/oncotarget.24547)
Supplement: Supplementary file 1 [file oncotarget-09-22546-s001.pdf]

Computational modeling of methionine cycle-based metabolism and DNA methylation and the implications for anti-cancer drug response prediction

SUPPLEMENTARY MATERIALS

Supplementary Information 1: Manhattan plot for five drugs against 30 pathways from the model MCPM.

See Supplementary File 1

Supplementary Information 2: An input data format for AutoAnalyse.

See Supplementary File 2

Supplementary Information 3: Kinetic Laws from AutoAnalyse.

See Supplementary File 3

Supplementary Information 4: The XML of the model MCPM.

See Supplementary File 4
