# Supplementary material for: Computational modeling of methionine cycle-based metabolism and DNA methylation and the implications for anti-cancer drug response prediction
Source: Oncotarget. 2018 Feb 21;9(32):22546–58. doi: 10.18632/oncotarget.24547 (PMC5989406; doi:10.18632/oncotarget.24547)
Supplement: Supplementary file 2 [file oncotarget-09-22546-s002.docx]

Supplementary Information 1: Manhattan plot for five drugs against 30 pathways from the model MCPM

The following listings present p-value and Spearman Correlation for all proteins ordered by the 30 pathways which are significant after Bonferroni adjustment (1239 proteins studied, p<4.035513e-05).

The lists are given for each of the 5 drugs.

Pathways used in the simulated data and corresponding numbers of metabolites:

D.1 D.2 D.3 D.4 D.5

Alanine_aspartate_and_glutamate_metabolism_hum 209 209 209 209 209

Amino_sugar_a_Nucleotide_sugar_hum 211 211 211 211 211

Arginine_biosynthesis_hum 124 124 124 124 124

Ascorbate_and_aldarate_metabolism_hum 88 88 88 88 88

Butanoate_metabolism_hum 153 153 153 153 153

Citrate_cycle_(TCA_cycle)_hum 160 160 160 160 160

Cysteine_and_methionine_metabolism_hum 250 250 250 250 250

Fatty_acid_biosynthesis_hum 106 106 106 106 106

Fatty_acid_degradation_hum 238 238 238 238 238

Fructose_and_mannose_metabolism_hum 163 163 163 163 163

Galactose_metabolism_hum 161 161 161 161 161

Glycerolipid_metabolism_hum 254 254 254 254 254

Glycine_serine_and_threonine_metabolism_hum 214 214 214 214 214

Glycolysis__Gluconeogenesis_hum 304 304 304 304 304

Glyoxylate_and_dicarboxylate_metabolism_hum 154 154 154 154 154

Histidine_metabolism_hum 136 136 136 136 136

Inositol_phosphate_metabolism_hum 284 284 284 284 284

Lysine_biosynthesis_hum 25 25 25 25 25

Nicotinate_and_nicotinamide_metabolism_hum 135 135 135 135 135

Pentose_and_glucuronate_interconversions_hum 125 125 125 125 125

Pentose_phosphate_pathway_hum 151 151 151 151 151

Propanoate_metabolism_hum 163 163 163 163 163

Purine_metabolism_hum 1046 1046 1046 1046 1046

Pyrimidine_metabolism_hum 398 398 398 398 398

Pyruvate_metabolism_hum 223 223 223 223 223

Starch_and_sucrose_metabolism_hum 217 217 217 217 217

Tyrosine_metabolism_hum 206 206 206 206 206

Valine_leucine_and_isoleucine_biosynthesis_hum 29 29 29 29 29

Valine_leucine_and_isoleucine_degradation_hum 238 238 238 238 238

Valine_leucine_isoleucine_degradation_hum 53 53 53 53 53

# “17-AAG"

$Alanine_aspartate_and_glutamate_metabolism_hum

estimate p.value

ASL.1_protein -0.2009363 1.065164e-05

NAALADL1.1_protein 0.1835330 5.940183e-05

$Amino_sugar_a_Nucleotide_sugar_hum

estimate p.value

UGDH.1_protein -0.2082336 4.948601e-06

GNPNAT1.1_protein -0.2367175 1.900091e-07

$Arginine_biosynthesis_hum

estimate p.value

ASL.1_protein -0.2009363 1.065164e-05

$Ascorbate_and_aldarate_metabolism_hum

estimate p.value

ALDH3A2.1_protein -0.2607255 8.651880e-09

ALDH9A1.1_protein -0.1843048 5.522109e-05

UGDH.1_protein -0.2082336 4.948601e-06

$Butanoate_metabolism_hum

estimate p.value

ECHS1.1_protein -0.1936343 2.231977e-05

$`Citrate_cycle_(TCA_cycle)_hum`

estimate p.value

IDH1.1_protein -0.1999937 1.173685e-05

OGDHL.1_protein 0.2066524 5.856462e-06

$Cysteine_and_methionine_metabolism_hum

estimate p.value

TST.1_protein -0.2224799 1.022805e-06

BCAT1.1_protein 0.2242975 8.300330e-07

GCLC.1_protein -0.2353503 2.244041e-07

$Fatty_acid_biosynthesis_hum

estimate p.value

ACSL3.2_protein -0.2389531 1.44434e-07

$Fatty_acid_degradation_hum

estimate p.value

ECHS1.1_protein -0.1936343 2.231977e-05

ALDH3A2.1_protein -0.2607255 8.651880e-09

ALDH9A1.1_protein -0.1843048 5.522109e-05

ALDH3A1.1_protein -0.2066428 5.862452e-06

ACADS.1_protein -0.2132433 2.877261e-06

$Fructose_and_mannose_metabolism_hum

estimate p.value

$Galactose_metabolism_hum

estimate p.value

$Glycerolipid_metabolism_hum

estimate p.value

ALDH3A2.1_protein -0.2607255 8.651880e-09

ALDH9A1.1_protein -0.1843048 5.522109e-05

AGPAT9.3_protein -0.1952184 1.905437e-05

PPAP2C.1_protein -0.2462907 5.759843e-08

AGPAT4.1_protein 0.2100974 4.050654e-06

$Glycine_serine_and_threonine_metabolism_hum

estimate p.value

$Glycolysis__Gluconeogenesis_hum

estimate p.value

ALDH3A2.1_protein -0.2607255 8.651880e-09

ALDH9A1.1_protein -0.1843048 5.522109e-05

ALDH3A1.1_protein -0.2066428 5.862452e-06

$Glyoxylate_and_dicarboxylate_metabolism_hum

estimate p.value

$Histidine_metabolism_hum

estimate p.value

ALDH3A2.1_protein -0.2607255 8.651880e-09

ALDH9A1.1_protein -0.1843048 5.522109e-05

ALDH3A1.1_protein -0.2066428 5.862452e-06

$Inositol_phosphate_metabolism_hum

estimate p.value

INPP1.1_protein -0.2046341 7.247622e-06

PIK3CD.1_protein 0.2067961 5.767777e-06

$Lysine_biosynthesis_hum

estimate p.value

$Nicotinate_and_nicotinamide_metabolism_hum

estimate p.value

NT5M.6_protein 0.1873568 4.125696e-05

$Pentose_and_glucuronate_interconversions_hum

estimate p.value

ALDH3A2.1_protein -0.2607255 8.651880e-09

ALDH9A1.1_protein -0.1843048 5.522109e-05

$Pentose_phosphate_pathway_hum

estimate p.value

G6PD.1_protein -0.1901484 3.147124e-05

$Propanoate_metabolism_hum

estimate p.value

ECHS1.1_protein -0.1936343 2.231977e-05

$Purine_metabolism_hum

estimate p.value

ATP6V1G2.3_protein 0.1892267 3.442930e-05

ATP6V1H.3_protein -0.1911549 2.851709e-05

NT5M.6_protein 0.1873568 4.125696e-05

$Pyrimidine_metabolism_hum

estimate p.value

NT5M.6_protein 0.1873568 4.125696e-05

ZCCHC11.1_protein 0.1992545 1.266078e-05

ZCCHC6.4_protein 0.1992545 1.266078e-05

$Pyruvate_metabolism_hum

estimate p.value

ALDH3A2.1_protein -0.2607255 8.651880e-09

ALDH9A1.1_protein -0.1843048 5.522109e-05

ALDH3A1.1_protein -0.2066428 5.862452e-06

$Starch_and_sucrose_metabolism_hum

estimate p.value

GPI.3_protein -0.1911549 2.851709e-05

UGDH.1_protein -0.2082336 4.948601e-06

$Tyrosine_metabolism_hum

estimate p.value

ALDH3A1.1_protein -0.2066428 5.862452e-06

$Valine_leucine_and_isoleucine_biosynthesis_hum

estimate p.value

BCAT1.1_protein 0.2242975 8.30033e-07

$Valine_leucine_and_isoleucine_degradation_hum

estimate p.value

ECHS1.1_protein -0.1936343 2.231977e-05

ALDH3A2.1_protein -0.2607255 8.651880e-09

ALDH9A1.1_protein -0.1843048 5.522109e-05

$Valine_leucine_isoleucine_degradation_hum

estimate p.value

BCAT1.1_protein 0.2242975 8.300330e-07

ECHS1.1_protein -0.1936343 2.231977e-05

# “Irinotecan"

$Alanine_aspartate_and_glutamate_metabolism_hum

estimate p.value

ALDH5A1.1_protein -0.2555877 9.093732e-06

PPAT.1_protein -0.3109387 5.200263e-08

$Amino_sugar_a_Nucleotide_sugar_hum

estimate p.value

PGM2.1_protein -0.2372187 3.975016e-05

HKDC1.1_protein 0.2491204 1.548636e-05

UGDH.1_protein 0.2316511 6.078448e-05

UXS1.1_protein 0.2812042 9.566846e-07

HEXA.1_protein 0.2693942 2.780599e-06

GNPDA1.1_protein 0.2628760 4.905516e-06

GALE.1_protein 0.3383264 2.635326e-09

$Arginine_biosynthesis_hum

estimate p.value

$Ascorbate_and_aldarate_metabolism_hum

estimate p.value

UGDH.1_protein 0.2316511 6.078448e-05

$Butanoate_metabolism_hum

estimate p.value

HADH.1_protein -0.3428741 1.560020e-09

ACADM.1_protein -0.2861624 6.021999e-07

OXCT1.1_protein -0.2659456 3.761700e-06

ALDH5A1.1_protein -0.2555877 9.093732e-06

$`Citrate_cycle_(TCA_cycle)_hum`

estimate p.value

$Cysteine_and_methionine_metabolism_hum

estimate p.value

LDHAL6B.1_protein -0.2478248 1.719974e-05

LDHB.1_protein -0.2531051 1.117454e-05

LDHC.2_protein -0.2332976 5.623029e-05

DNMT1.1_protein -0.3677740 7.581825e-11

TRDMT1.1_protein -0.2585864 7.607665e-06

$Fatty_acid_biosynthesis_hum

estimate p.value

$Fatty_acid_degradation_hum

estimate p.value

HADH.1_protein -0.3428741 1.560020e-09

ACADM.1_protein -0.2861624 6.021999e-07

ALDH3B1.1_protein 0.2886480 5.355014e-07

GCDH.2_protein -0.2357712 4.443507e-05

$Fructose_and_mannose_metabolism_hum

estimate p.value

HKDC1.1_protein 0.2491204 1.548636e-05

PFKFB2.1_protein 0.3330789 4.775587e-09

$Galactose_metabolism_hum

estimate p.value

GLB1.1_protein 0.2841361 7.283950e-07

PGM2.1_protein -0.2372187 3.975016e-05

HKDC1.1_protein 0.2491204 1.548636e-05

GALE.2_protein 0.3383264 2.635326e-09

$Glycerolipid_metabolism_hum

estimate p.value

AGPAT9.3_protein 0.3000552 1.568846e-07

ABHD12.3_protein 0.3673314 8.019052e-11

PPAP2B.1_protein 0.2998311 1.843363e-07

DGKE.1_protein -0.2966082 2.517930e-07

PNPLA3.1_protein 0.2536232 1.070609e-05

$Glycine_serine_and_threonine_metabolism_hum

estimate p.value

MAOA.1_protein 0.2452153 2.121086e-05

SHMT2.2_protein -0.2748395 1.710717e-06

$Glycolysis__Gluconeogenesis_hum

estimate p.value

ALDH3B1.1_protein 0.2886480 5.355014e-07

ACSS2.4_protein 0.2647413 4.529545e-06

LDHB.1_protein -0.2531051 1.117454e-05

LDHAL6B.1_protein -0.2476843 1.848501e-05

PGM2.1_protein -0.2372187 3.975016e-05

HKDC1.1_protein 0.2491204 1.548636e-05

GPI.3_protein -0.2535129 1.155552e-05

$Glyoxylate_and_dicarboxylate_metabolism_hum

estimate p.value

SHMT2.2_protein -0.2748395 1.710717e-06

$Histidine_metabolism_hum

estimate p.value

ALDH3B1.1_protein 0.2886480 5.355014e-07

MAOA.1_protein 0.2452153 2.121086e-05

CNDP2.1_protein -0.2596799 6.444921e-06

$Inositol_phosphate_metabolism_hum

estimate p.value

PTEN.1_protein -0.2331404 5.689735e-05

CDIPT.1_protein 0.2934698 2.994666e-07

PI4K2A.1_protein 0.2375979 3.860235e-05

PLCB4.1_protein 0.2582767 7.257260e-06

PLCD3.1_protein 0.3704671 7.404685e-11

INPP4B.1_protein 0.2554495 9.199120e-06

INPP5B.2_protein -0.2569198 8.134765e-06

PIK3CG.1_protein -0.2830096 8.091335e-07

PIP4K2C.1_protein 0.3562168 3.188388e-10

$Lysine_biosynthesis_hum

estimate p.value

$Nicotinate_and_nicotinamide_metabolism_hum

estimate p.value

NT5E.1_protein 0.2553026 9.980934e-06

ENPP1.1_protein 0.2361915 4.302352e-05

PNP.1_protein -0.2596571 6.457422e-06

$Pentose_and_glucuronate_interconversions_hum

estimate p.value

$Pentose_phosphate_pathway_hum

estimate p.value

PGM2.1_protein -0.2372187 3.975016e-05

RPIA.1_protein -0.3694813 6.102231e-11

$Propanoate_metabolism_hum

estimate p.value

ACADM.1_protein -0.2861624 6.021999e-07

ACSS2.4_protein 0.2647413 4.529545e-06

LDHAL6B.1_protein -0.2478248 1.719974e-05

LDHB.1_protein -0.2531051 1.117454e-05

BCKDHA.1_protein -0.2461402 1.969729e-05

$Purine_metabolism_hum

estimate p.value

NT5E.1_protein 0.2553026 9.980934e-06

PGM2.1_protein -0.2372187 3.975016e-05

ENPP1.1_protein 0.2361915 4.302352e-05

EEF2.2_protein -0.3002222 1.543005e-07

PAPSS2.1_protein 0.2426179 2.607268e-05

ATP9A.2_protein 0.2512921 1.297185e-05

ATP5F1.2_protein -0.2341333 5.036292e-05

ATP5A1.1_protein -0.2809501 9.794163e-07

ATP6V0C.1_protein 0.2625098 5.062268e-06

ATP2A1.1_protein -0.2412303 2.908435e-05

ATP5B.1_protein -0.2509617 1.332761e-05

ATP8B1.1_protein 0.2644089 4.659700e-06

SPAST.2_protein -0.3387935 2.498123e-09

ATP6V0A2.1_protein -0.2633292 4.717931e-06

KATNA1.1_protein -0.3270065 9.371440e-09

ATP6AP1.1_protein 0.2577623 7.578723e-06

ATP6V1B1.1_protein 0.2364676 4.211972e-05

ATP6V0E1.1_protein 0.4056839 4.483729e-13

ADCY9.1_protein 0.2974005 2.332887e-07

ADCY6.1_protein 0.3115568 5.745343e-08

NUDT2.2_protein -0.2612776 6.073772e-06

DCK.2_protein -0.4013045 8.391595e-13

ENTPD6.1_protein 0.2526207 1.163007e-05

POLD1.2_protein -0.3286959 7.780285e-09

POLE.1_protein -0.3183771 2.381964e-08

POLE2.1_protein -0.2480447 1.689675e-05

POLE3.2_protein -0.3215406 1.697837e-08

POLQ.1_protein -0.3206421 1.869957e-08

POLA1.1_protein -0.3510353 7.784758e-10

POLR2A.1_protein -0.2856091 6.344055e-07

POLR1B.1_protein -0.3350290 3.833924e-09

PAPOLG.1_protein -0.3245184 1.230051e-08

PPAT.1_protein -0.3109387 5.200263e-08

IMPDH2.1_protein -0.2464716 1.918038e-05

PNP.1_protein -0.2596571 6.457422e-06

RRM2.1_protein -0.3427313 1.586113e-09

RRM1.1_protein -0.2941942 2.791312e-07

PAICS.1_protein -0.3268226 9.562513e-09

PFAS.1_protein -0.2872689 5.424355e-07

$Pyrimidine_metabolism_hum

estimate p.value

NT5E.1_protein 0.2553026 9.980934e-06

DCK.2_protein -0.4013045 8.391595e-13

ENTPD6.1_protein 0.2526207 1.163007e-05

POLD1.2_protein -0.3286959 7.780285e-09

POLE.1_protein -0.3183771 2.381964e-08

POLE2.1_protein -0.2480447 1.689675e-05

POLE3.2_protein -0.3215406 1.697837e-08

POLQ.1_protein -0.3206421 1.869957e-08

POLA1.1_protein -0.3510353 7.784758e-10

POLR2A.1_protein -0.2856091 6.344055e-07

POLR1B.1_protein -0.3350290 3.833924e-09

PNP.1_protein -0.2596571 6.457422e-06

RRM2.1_protein -0.3427313 1.586113e-09

RRM1.1_protein -0.2941942 2.791312e-07

ZCCHC11.1_protein -0.2477996 1.723475e-05

ZCCHC6.4_protein -0.2477996 1.723475e-05

UCK2.1_protein -0.2739618 1.851358e-06

TK2.1_protein 0.2749338 1.696227e-06

DHODH.1_protein -0.2937925 2.902387e-07

UPP1.1_protein 0.2975335 2.013357e-07

TXNRD2.5_protein -0.3768917 3.264923e-11

TXNRD1.1_protein -0.3711745 6.773718e-11

DUT.2_protein -0.3683558 7.042131e-11

TYMS.1_protein -0.2342784 4.980918e-05

$Pyruvate_metabolism_hum

estimate p.value

ALDH3B1.1_protein 0.2886480 5.355014e-07

LDHAL6B.1_protein -0.2478248 1.719974e-05

LDHB.1_protein -0.2531051 1.117454e-05

LDHC.2_protein -0.2476843 1.848501e-05

ME2.1_protein -0.3218106 1.649167e-08

$Starch_and_sucrose_metabolism_hum

estimate p.value

PGM2.1_protein -0.2372187 3.975016e-05

HKDC1.1_protein 0.2491204 1.548636e-05

UGDH.1_protein 0.2316511 6.078448e-05

ENPP1.1_protein 0.2361915 4.302352e-05

UXS1.1_protein 0.2812042 9.566846e-07

PYGB.1_protein 0.3458112 1.106886e-09

$Tyrosine_metabolism_hum

estimate p.value

ALDH3B1.1_protein 0.2886480 5.355014e-07

MAOA.1_protein 0.2452153 2.121086e-05

$Valine_leucine_and_isoleucine_biosynthesis_hum

estimate p.value

$Valine_leucine_and_isoleucine_degradation_hum

estimate p.value

HADH.1_protein -0.3428741 1.560020e-09

ACADM.1_protein -0.2861624 6.021999e-07

OXCT1.1_protein -0.2659456 3.761700e-06

HIBADH.1_protein 0.2599491 6.299349e-06

$Valine_leucine_isoleucine_degradation_hum

estimate p.value

# “Paclitaxel"

$Alanine_aspartate_and_glutamate_metabolism_hum

estimate p.value

$Amino_sugar_a_Nucleotide_sugar_hum

estimate p.value

$Arginine_biosynthesis_hum

estimate p.value

$Ascorbate_and_aldarate_metabolism_hum

estimate p.value

$Butanoate_metabolism_hum

estimate p.value

$`Citrate_cycle_(TCA_cycle)_hum`

estimate p.value

$Cysteine_and_methionine_metabolism_hum

estimate p.value

$Fatty_acid_biosynthesis_hum

estimate p.value

$Fatty_acid_degradation_hum

estimate p.value

$Fructose_and_mannose_metabolism_hum

estimate p.value

$Galactose_metabolism_hum

estimate p.value

$Glycerolipid_metabolism_hum

estimate p.value

$Glycine_serine_and_threonine_metabolism_hum

estimate p.value

$Glycolysis__Gluconeogenesis_hum

estimate p.value

$Glyoxylate_and_dicarboxylate_metabolism_hum

estimate p.value

$Histidine_metabolism_hum

estimate p.value

$Inositol_phosphate_metabolism_hum

estimate p.value

$Lysine_biosynthesis_hum

estimate p.value

$Nicotinate_and_nicotinamide_metabolism_hum

estimate p.value

$Pentose_and_glucuronate_interconversions_hum

estimate p.value

$Pentose_phosphate_pathway_hum

estimate p.value

$Propanoate_metabolism_hum

estimate p.value

$Purine_metabolism_hum

estimate p.value

$Pyrimidine_metabolism_hum

estimate p.value

DUT.2_protein -0.185852 4.766235e-05

$Pyruvate_metabolism_hum

estimate p.value

$Starch_and_sucrose_metabolism_hum

estimate p.value

PYGB.1_protein 0.19837 1.385724e-05

$Tyrosine_metabolism_hum

estimate p.value

$Valine_leucine_and_isoleucine_biosynthesis_hum

estimate p.value

$Valine_leucine_and_isoleucine_degradation_hum

estimate p.value

$Valine_leucine_isoleucine_degradation_hum

estimate p.value

# “Panobinostat"

$Alanine_aspartate_and_glutamate_metabolism_hum

estimate p.value

GAD1.2_protein 0.1899536 3.512055e-05

ALDH5A1.1_protein -0.2962646 5.622042e-11

CAD.1_protein -0.2092794 4.745085e-06

PPAT.1_protein -0.3136560 3.447744e-12

TNRC6B.1_protein -0.2686951 3.241988e-09

GFPT2.1_protein 0.3197027 1.250209e-12

NAT8L.2_protein -0.2823715 5.474211e-10

$Amino_sugar_a_Nucleotide_sugar_hum

estimate p.value

PGM1.1_protein 0.2839040 3.658780e-10

HKDC1.1_protein 0.2023872 9.787843e-06

UGDH.1_protein 0.2259442 7.442040e-07

GMPPA.1_protein 0.1950380 2.062416e-05

GMPPB.1_protein -0.2033654 8.845084e-06

GALK1.1_protein -0.2076632 5.635465e-06

UXS1.1_protein 0.1925479 2.638504e-05

GFPT2.1_protein 0.3197027 1.250209e-12

RENBP.1_protein -0.1995267 1.312474e-05

GALE.1_protein 0.2370421 1.997682e-07

$Arginine_biosynthesis_hum

estimate p.value

CAD.1_protein -0.2092794 4.745085e-06

$Ascorbate_and_aldarate_metabolism_hum

estimate p.value

ALDH3A2.1_protein 0.1991597 1.362405e-05

ALDH7A1.1_protein 0.1836315 6.213878e-05

UGDH.1_protein 0.2259442 7.442040e-07

$Butanoate_metabolism_hum

estimate p.value

ACAT1.1_protein -0.1960827 1.858186e-05

HADHA.1_protein -0.2836503 3.798566e-10

HADH.1_protein -0.3631526 4.249154e-16

ACADM.1_protein -0.2882187 1.922505e-10

BDH1.1_protein -0.2434250 9.098464e-08

GAD1.2_protein 0.1899536 3.512055e-05

ALDH5A1.1_protein -0.2962646 5.622042e-11

L2HGDH.1_protein -0.1993156 1.340989e-05

$`Citrate_cycle_(TCA_cycle)_hum`

estimate p.value

DLD.1_protein -0.2236823 9.651764e-07

IDH2.1_protein -0.1847019 5.618426e-05

PC.2_protein -0.1982117 1.560031e-05

ACLY.1_protein -0.2611071 9.161585e-09

ACO2.1_protein -0.2079798 5.449375e-06

$Cysteine_and_methionine_metabolism_hum

estimate p.value

LDHAL6B.1_protein -0.2012333 1.102295e-05

MTR.1_protein -0.1966665 1.752572e-05

MPST.1_protein -0.2177760 1.878995e-06

DNMT1.1_protein -0.2616525 8.511669e-09

ENOPH1.1_protein -0.2001912 1.226469e-05

GSS.1_protein 0.2031634 9.032427e-06

$Fatty_acid_biosynthesis_hum

estimate p.value

$Fatty_acid_degradation_hum

estimate p.value

ACAA2.1_protein -0.2503146 3.797014e-08

HADHA.1_protein -0.2836503 3.798566e-10

HADH.1_protein -0.3631526 4.249154e-16

ACADM.1_protein -0.2882187 1.922505e-10

CPT2.1_protein -0.2103031 4.252403e-06

ACAT1.1_protein -0.1875362 4.425728e-05

ALDH1A3.1_protein 0.3818062 9.274430e-18

ALDH3B1.1_protein 0.3181003 1.639458e-12

ALDH3A2.1_protein 0.1991597 1.362405e-05

ALDH7A1.1_protein 0.1836315 6.213878e-05

GCDH.2_protein -0.2132268 3.099974e-06

$Fructose_and_mannose_metabolism_hum

estimate p.value

HKDC1.1_protein 0.2023872 9.787843e-06

PFKP.1_protein 0.1859430 4.995509e-05

KHK.1_protein -0.2378249 1.816160e-07

GMPPA.1_protein 0.1950380 2.062416e-05

GMPPB.1_protein -0.2033654 8.845084e-06

$Galactose_metabolism_hum

estimate p.value

PGM1.1_protein 0.2839040 3.658780e-10

HKDC1.1_protein 0.2023872 9.787843e-06

PFKP.1_protein 0.1859430 4.995509e-05

GALE.2_protein 0.2370421 1.997682e-07

B4GALT1.1_protein 0.2231898 1.021028e-06

GANC.1_protein -0.1950543 2.059089e-05

GALK1.1_protein -0.2076632 5.635465e-06

$Glycerolipid_metabolism_hum

estimate p.value

ALDH3A2.1_protein 0.1991597 1.362405e-05

ALDH7A1.1_protein 0.1836315 6.213878e-05

GK.1_protein -0.2948301 7.019846e-11

AGPAT9.3_protein 0.3170172 1.967309e-12

ABHD12.3_protein 0.2461452 6.463594e-08

MGLL.1_protein 0.2396672 1.449485e-07

PPAP2C.1_protein 0.2616466 8.518447e-09

PPAP2B.1_protein 0.2901631 1.433377e-10

PPAP2A.2_protein 0.2309273 4.156943e-07

DGKE.1_protein -0.2474712 5.463227e-08

MBOAT2.1_protein 0.2218724 1.186079e-06

$Glycine_serine_and_threonine_metabolism_hum

estimate p.value

ALDH7A1.1_protein 0.1836315 6.213878e-05

DLD.1_protein -0.2236823 9.651764e-07

GAMT.1_protein -0.1958491 1.902126e-05

SHMT2.2_protein -0.2606595 9.730648e-09

$Glycolysis__Gluconeogenesis_hum

estimate p.value

GAPDH.1_protein 0.1865920 4.696304e-05

ENO1.1_protein 0.1865920 4.696304e-05

PKM2.3_protein 0.2409805 1.232848e-07

ALDH1A3.1_protein 0.3818062 9.274430e-18

ALDH3B1.1_protein 0.3181003 1.639458e-12

ALDH3A2.1_protein 0.1991597 1.362405e-05

ALDH7A1.1_protein 0.1836315 6.213878e-05

PKLR.2_protein -0.1982117 1.560031e-05

ACSS1.1_protein -0.1868714 4.572831e-05

ACSS2.4_protein 0.2028770 9.304485e-06

ENO3.1_protein -0.2554274 1.951842e-08

PGM1.1_protein 0.2839040 3.658780e-10

BPGM.2_protein 0.1863269 4.963168e-05

HKDC1.1_protein 0.2023872 9.787843e-06

GPI.3_protein -0.2363772 2.354829e-07

PFKP.1_protein 0.1859430 4.995509e-05

DLD.1_protein -0.2236823 9.651764e-07

ADPGK.1_protein -0.2261489 7.268069e-07

$Glyoxylate_and_dicarboxylate_metabolism_hum

estimate p.value

ACAT1.1_protein -0.1960827 1.858186e-05

DLD.1_protein -0.2236823 9.651764e-07

ACO2.1_protein -0.2079798 5.449375e-06

HYI.2_protein 0.2934255 8.714274e-11

PCCB.1_protein -0.2574386 1.496339e-08

AFMID.1_protein -0.2740598 1.524877e-09

MCEE.1_protein -0.1878790 4.152421e-05

SHMT2.2_protein -0.2606595 9.730648e-09

$Histidine_metabolism_hum

estimate p.value

ALDH1A3.1_protein 0.3818062 9.274430e-18

ALDH3B1.1_protein 0.3181003 1.639458e-12

ALDH3A2.1_protein 0.1991597 1.362405e-05

ALDH7A1.1_protein 0.1836315 6.213878e-05

DDC.1_protein -0.2335791 3.032716e-07

CNDP2.1_protein -0.2716598 2.141252e-09

$Inositol_phosphate_metabolism_hum

estimate p.value

PTEN.1_protein -0.2559013 1.833722e-08

CDIPT.1_protein 0.2011008 1.117400e-05

PI4K2A.1_protein 0.1853396 5.289724e-05

TOP3B.2_protein -0.2087335 5.029596e-06

PLCB4.1_protein 0.2088310 4.977643e-06

PLCD3.1_protein 0.3062271 1.162129e-11

PIP5K1B.1_protein -0.2800484 6.442428e-10

INPP4B.1_protein 0.2108777 3.997652e-06

INPP5B.2_protein -0.2889699 1.716809e-10

SYNJ2.1_protein 0.2452952 7.195516e-08

PIK3CG.1_protein -0.2241361 9.163247e-07

PIK3C2B.1_protein -0.2464895 6.188009e-08

PIP5K2B.1_protein -0.2093815 4.693566e-06

PIP4K2C.1_protein 0.1969107 1.710111e-05

INPP5D.1_protein -0.2975997 4.567304e-11

$Lysine_biosynthesis_hum

estimate p.value

ALDH7A1.1_protein 0.1836315 6.213878e-05

$Nicotinate_and_nicotinamide_metabolism_hum

estimate p.value

NT5E.1_protein 0.3049326 1.431208e-11

AOX1.1_protein 0.2366731 2.272890e-07

CD38.1_protein -0.1913490 2.967462e-05

NADK.1_protein -0.2291017 5.153458e-07

NMNAT2.1_protein 0.1955255 1.964596e-05

NNMT.1_protein 0.3829126 7.334179e-18

NAPRT1.5_protein -0.2215979 1.223555e-06

$Pentose_and_glucuronate_interconversions_hum

estimate p.value

ALDH3A2.1_protein 0.1991597 1.362405e-05

ALDH7A1.1_protein 0.1836315 6.213878e-05

XYLB.1_protein -0.2504954 3.709661e-08

DCXR.1_protein -0.2695951 2.859928e-09

$Pentose_phosphate_pathway_hum

estimate p.value

PGM1.1_protein 0.2839040 3.658780e-10

PFKP.1_protein 0.1859430 4.995509e-05

RPIA.1_protein -0.3535799 2.751471e-15

G6PD.1_protein 0.2869358 2.330558e-10

$Propanoate_metabolism_hum

estimate p.value

HADHA.1_protein -0.2836503 3.798566e-10

ACAT1.1_protein -0.2688100 3.685937e-09

ACADM.1_protein -0.2882187 1.922505e-10

ACSS1.1_protein -0.1868714 4.572831e-05

ACSS2.4_protein 0.2028770 9.304485e-06

LDHAL6B.1_protein -0.2012333 1.102295e-05

DLD.1_protein -0.2236823 9.651764e-07

MCEE.1_protein -0.1878790 4.152421e-05

BCKDHB.1_protein -0.2195653 1.538584e-06

BCKDHA.1_protein -0.2392027 1.534563e-07

$Purine_metabolism_hum

estimate p.value

PKM2.3_protein 0.2409805 1.232848e-07

NT5E.1_protein 0.3049326 1.431208e-11

PKLR.2_protein -0.1982117 1.560031e-05

PGM1.1_protein 0.2839040 3.658780e-10

EEF2.2_protein -0.2605204 9.914455e-09

PAPSS1.2_protein -0.1975198 1.608428e-05

PAPSS2.1_protein 0.3375621 5.454525e-14

ATP9A.2_protein 0.2327958 3.330039e-07

ATP1A3.1_protein -0.1907901 3.133737e-05

ATP5A1.1_protein -0.3139063 3.307492e-12

ATP9B.1_protein -0.2563337 1.732025e-08

ENTPD1.2_protein -0.2047273 7.675550e-06

ATP10D.1_protein 0.2227778 1.070121e-06

ATP2A1.1_protein -0.1904629 3.235142e-05

ATP5B.1_protein -0.2639573 6.225467e-09

ATP8B1.1_protein 0.2971300 4.914314e-11

ATP5G2.1_protein -0.2524711 2.872892e-08

ATP8A1.1_protein -0.2974654 4.664041e-11

ATP2A3.1_protein -0.3226844 7.517245e-13

SPAST.2_protein -0.2680800 3.531116e-09

ATP6V0A2.1_protein -0.3757396 3.306111e-17

ATP6V1G2.3_protein -0.2590737 1.203575e-08

TCIRG1.1_protein 0.1875111 4.301500e-05

ATP6V1B2.1_protein -0.2248815 8.411805e-07

ATP6V0E1.1_protein 0.3775625 2.262852e-17

ATP6V1D.1_protein 0.3028840 1.985734e-11

CFTR.2_protein 0.3028840 1.985734e-11

ADCY9.1_protein 0.2028248 9.354814e-06

AK5.1_protein 0.1941008 2.263610e-05

DCK.2_protein -0.3492373 6.289561e-15

FHIT.1_protein -0.2122358 3.452284e-06

PDE8A.1_protein 0.1854857 5.217029e-05

PDE4A.1_protein -0.2155785 2.396319e-06

PDE3B.1_protein -0.3302608 2.012008e-13

GFM1.1_protein -0.1919652 2.793808e-05

TUFM.1_protein -0.2326346 3.394624e-07

EEF1A1.1_protein -0.1932636 2.458963e-05

POLD1.2_protein -0.3008324 2.749432e-11

POLE.1_protein -0.3634524 4.003538e-16

POLG.1_protein -0.2913695 1.193321e-10

POLE2.1_protein -0.2214219 1.248177e-06

POLE3.2_protein -0.2454014 7.099877e-08

REV3L.4_protein -0.1879231 4.134907e-05

POLH.1_protein -0.1911551 3.024171e-05

POLQ.1_protein -0.2320020 3.659913e-07

POLA1.1_protein -0.2415773 1.255340e-07

POLR2B.1_protein -0.2173349 1.973410e-06

POLR1B.1_protein -0.3190406 1.398647e-12

PAPD4.3_protein -0.2041401 8.160505e-06

PAPOLG.1_protein -0.3219189 8.570595e-13

POLR3B.1_protein -0.2429595 9.642765e-08

POLR3A.2_protein -0.2440792 8.383353e-08

PAPOLA.1_protein -0.2440792 8.383353e-08

PPAT.1_protein -0.3136560 3.447744e-12

TNRC6B.1_protein -0.2686951 3.241988e-09

IMPDH2.1_protein -0.1994596 1.321482e-05

GDA.1_protein 0.2074745 5.749290e-06

XDH.1_protein 0.2775500 9.252407e-10

RRM1.1_protein -0.1925645 2.634217e-05

PRUNE.4_protein -0.2899928 1.470841e-10

GART.3_protein -0.1847060 5.616217e-05

PAICS.1_protein -0.2600946 1.049796e-08

NME1.1_protein -0.2192443 1.594951e-06

VILL.1_protein -0.2242282 9.067006e-07

HDDC3.1_protein -0.2246546 8.634116e-07

$Pyrimidine_metabolism_hum

estimate p.value

NT5E.1_protein 0.3049326 1.431208e-11

CAD.1_protein -0.2092794 4.745085e-06

ENTPD1.2_protein -0.2047273 7.675550e-06

DCK.2_protein -0.3492373 6.289561e-15

POLD1.2_protein -0.3008324 2.749432e-11

POLE.1_protein -0.3634524 4.003538e-16

POLG.1_protein -0.2913695 1.193321e-10

POLE2.1_protein -0.2214219 1.248177e-06

POLE3.2_protein -0.2454014 7.099877e-08

REV3L.4_protein -0.1879231 4.134907e-05

POLH.1_protein -0.1911551 3.024171e-05

POLQ.1_protein -0.2320020 3.659913e-07

POLA1.1_protein -0.2415773 1.255340e-07

POLR2B.1_protein -0.2173349 1.973410e-06

POLR1B.1_protein -0.3190406 1.398647e-12

POLR3B.1_protein -0.2429595 9.642765e-08

POLR3A.2_protein -0.2440792 8.383353e-08

RRM1.1_protein -0.1925645 2.634217e-05

ZCCHC11.1_protein -0.2117950 3.620975e-06

ZCCHC6.4_protein -0.2117950 3.620975e-06

UCK2.1_protein -0.2967471 5.215998e-11

DPYD.1_protein 0.1889662 3.739964e-05

TK2.1_protein 0.2631288 6.968747e-09

DHODH.1_protein -0.2080260 5.422677e-06

UPP1.1_protein 0.3762676 2.963006e-17

TXNRD2.5_protein -0.1887307 3.949305e-05

TXNRD1.1_protein -0.2044674 8.249767e-06

DUT.2_protein -0.3050048 1.414711e-11

TYMS.1_protein -0.1883024 3.986915e-05

$Pyruvate_metabolism_hum

estimate p.value

PKM2.3_protein 0.2409805 1.232848e-07

ACAT1.1_protein -0.1960827 1.858186e-05

ALDH1A3.1_protein 0.3818062 9.274430e-18

ALDH3B1.1_protein 0.3181003 1.639458e-12

ALDH3A2.1_protein 0.1991597 1.362405e-05

ALDH7A1.1_protein 0.1836315 6.213878e-05

PKLR.2_protein -0.1982117 1.560031e-05

LDHAL6B.1_protein -0.2012333 1.102295e-05

DLD.1_protein -0.2236823 9.651764e-07

ME2.1_protein -0.2845930 3.303907e-10

ME1.1_protein 0.2420540 1.079307e-07

$Starch_and_sucrose_metabolism_hum

estimate p.value

PGM1.1_protein 0.2839040 3.658780e-10

HKDC1.1_protein 0.2023872 9.787843e-06

UGDH.1_protein 0.2259442 7.442040e-07

GANC.1_protein -0.1950543 2.059089e-05

UXS1.1_protein 0.1925479 2.638504e-05

GBE1.1_protein 0.1938226 2.326838e-05

PYGL.1_protein 0.1838956 6.061650e-05

PYGB.1_protein 0.3315424 1.604030e-13

$Tyrosine_metabolism_hum

estimate p.value

ALDH1A3.1_protein 0.3818062 9.274430e-18

ALDH3B1.1_protein 0.3181003 1.639458e-12

DDC.1_protein -0.2335791 3.032716e-07

AOX1.1_protein 0.2366731 2.272890e-07

$Valine_leucine_and_isoleucine_biosynthesis_hum

estimate p.value

$Valine_leucine_and_isoleucine_degradation_hum

estimate p.value

ACAT1.1_protein -0.1960827 1.858186e-05

ACAA2.1_protein -0.2503146 3.797014e-08

HADHA.1_protein -0.2836503 3.798566e-10

HADH.1_protein -0.3631526 4.249154e-16

ALDH3A2.1_protein 0.1991597 1.362405e-05

ALDH7A1.1_protein 0.1836315 6.213878e-05

ACADM.1_protein -0.2882187 1.922505e-10

DLD.1_protein -0.2236823 9.651764e-07

PCCB.1_protein -0.2574386 1.496339e-08

MCEE.1_protein -0.1878790 4.152421e-05

AOX1.1_protein -0.1871224 4.603097e-05

MCCC1.1_protein -0.2297893 4.753767e-07

HIBADH.1_protein 0.2029219 9.261262e-06

BCKDHB.1_protein -0.2195653 1.538584e-06

$Valine_leucine_isoleucine_degradation_hum

estimate p.value

ACAT1.1_protein -0.1960827 1.858186e-05

ACAA2.1_protein -0.2503146 3.797014e-08

# “Topotecan"

$Alanine_aspartate_and_glutamate_metabolism_hum

estimate p.value

ALDH5A1.1_protein -0.2301513 4.069494e-07

CAD.1_protein -0.2552310 1.743434e-08

PPAT.1_protein -0.2843346 2.888479e-10

ADSSL1.1_protein 0.1858081 4.698411e-05

ASL.1_protein 0.2174048 1.770405e-06

GFPT1.4_protein 0.1854203 4.875861e-05

$Amino_sugar_a_Nucleotide_sugar_hum

estimate p.value

PGM2.1_protein -0.2487261 4.080762e-08

UGDH.1_protein 0.2507144 3.154561e-08

UXS1.1_protein 0.2375562 1.664108e-07

GFPT1.4_protein 0.1854203 4.875861e-05

HEXA.1_protein 0.2246413 7.765697e-07

PGM3.1_protein 0.1914253 2.722937e-05

GALE.1_protein 0.2095294 4.206627e-06

$Arginine_biosynthesis_hum

estimate p.value

CAD.1_protein -0.2552310 1.743434e-08

ASL.1_protein 0.2174048 1.770405e-06

$Ascorbate_and_aldarate_metabolism_hum

estimate p.value

ALDH3A2.1_protein 0.2146441 2.406802e-06

UGDH.1_protein 0.2507144 3.154561e-08

$Butanoate_metabolism_hum

estimate p.value

ACAT1.1_protein -0.2019688 9.366219e-06

HADH.1_protein -0.2723732 1.653031e-09

ACADM.1_protein -0.3079106 7.222179e-12

OXCT1.1_protein -0.2768129 8.736265e-10

ALDH5A1.1_protein -0.2301513 4.069494e-07

L2HGDH.1_protein -0.2108777 3.635703e-06

$`Citrate_cycle_(TCA_cycle)_hum`

estimate p.value

IDH1.1_protein 0.2096817 4.138086e-06

PC.2_protein -0.2297486 4.268652e-07

ACLY.1_protein -0.2019515 9.383090e-06

$Cysteine_and_methionine_metabolism_hum

estimate p.value

LDHAL6B.1_protein -0.1893068 3.351193e-05

LDHB.1_protein -0.2690584 2.641359e-09

MAT2B.2_protein -0.2233612 9.002656e-07

MAT2A.1_protein -0.1940928 2.089800e-05

LDHC.2_protein -0.2122793 3.121013e-06

DNMT1.1_protein -0.3582623 8.414894e-16

TRDMT1.1_protein -0.2649549 4.677206e-09

BCAT1.1_protein -0.2014008 9.934887e-06

AGXT2L2.1_protein -0.2326568 3.017015e-07

$Fatty_acid_biosynthesis_hum

estimate p.value

$Fatty_acid_degradation_hum

estimate p.value

HADH.1_protein -0.2723732 1.653031e-09

ACADM.1_protein -0.3079106 7.222179e-12

ALDH1A3.1_protein 0.2120611 3.196291e-06

ALDH3B1.1_protein 0.2624636 6.585836e-09

ALDH3A2.1_protein 0.2146441 2.406802e-06

GCDH.2_protein -0.2690049 2.661254e-09

$Fructose_and_mannose_metabolism_hum

estimate p.value

KHK.1_protein -0.1846220 5.261320e-05

PFKFB2.1_protein 0.2842732 2.915058e-10

$Galactose_metabolism_hum

estimate p.value

GLB1.1_protein 0.2206281 1.230695e-06

PGM2.1_protein -0.2487261 4.080762e-08

GALE.2_protein 0.2095294 4.206627e-06

B4GALT1.1_protein 0.1880052 3.802850e-05

GANC.1_protein -0.2472651 4.923646e-08

$Glycerolipid_metabolism_hum

estimate p.value

ALDH3A2.1_protein 0.2146441 2.406802e-06

AGPAT9.3_protein 0.3052339 1.116778e-11

ABHD12.3_protein 0.3018117 1.937260e-11

PPAP2C.1_protein 0.2251439 7.326122e-07

PPAP2B.1_protein 0.3243139 4.528774e-13

DGKE.1_protein -0.2475447 4.750328e-08

$Glycine_serine_and_threonine_metabolism_hum

estimate p.value

MAOA.1_protein 0.2038570 7.690468e-06

SHMT2.2_protein -0.2731805 1.473309e-09

SDSL.1_protein 0.2342948 2.476403e-07

$Glycolysis__Gluconeogenesis_hum

estimate p.value

ALDH1A3.1_protein 0.2120611 3.196291e-06

ALDH3B1.1_protein 0.2624636 6.585836e-09

ALDH3A2.1_protein 0.2146441 2.406802e-06

PKLR.2_protein -0.2297486 4.268652e-07

ACSS2.4_protein 0.2396906 1.278907e-07

LDHB.1_protein -0.2690584 2.641359e-09

LDHAL6B.1_protein -0.2410739 1.076849e-07

PGM2.1_protein -0.2487261 4.080762e-08

GPI.3_protein -0.2285009 4.946993e-07

$Glyoxylate_and_dicarboxylate_metabolism_hum

estimate p.value

ACAT1.1_protein -0.2019688 9.366219e-06

AFMID.1_protein -0.1984182 1.350203e-05

SHMT2.2_protein -0.2731805 1.473309e-09

$Histidine_metabolism_hum

estimate p.value

ALDH1A3.1_protein 0.2120611 3.196291e-06

ALDH3B1.1_protein 0.2624636 6.585836e-09

ALDH3A2.1_protein 0.2146441 2.406802e-06

HNMT.1_protein 0.1846151 5.264743e-05

MAOA.1_protein 0.2038570 7.690468e-06

C9orf41.1_protein -0.2440661 7.396768e-08

CNDP2.1_protein -0.2227410 9.667917e-07

$Inositol_phosphate_metabolism_hum

estimate p.value

PTEN.1_protein -0.2219005 1.064518e-06

CDIPT.1_protein 0.3018320 1.931001e-11

PI4K2B.4_protein -0.2159293 2.087222e-06

TOP3B.2_protein -0.2086889 4.604874e-06

ITPKB.1_protein -0.1993476 1.227709e-05

PLCB4.1_protein 0.2493081 3.785437e-08

PLCD3.1_protein 0.2496708 3.611933e-08

INPP4B.1_protein 0.2297141 4.286098e-07

INPP5B.2_protein -0.3369640 4.756477e-14

OCRL.2_protein 0.2140405 2.572574e-06

PIK3CG.1_protein -0.2421315 9.435139e-08

PIK3CD.1_protein -0.2049284 6.870972e-06

PIKFYVE.2_protein -0.2047285 7.017204e-06

PIP4K2C.1_protein 0.3369385 4.778644e-14

INPP5D.1_protein -0.2529843 2.344945e-08

$Lysine_biosynthesis_hum

estimate p.value

$Nicotinate_and_nicotinamide_metabolism_hum

estimate p.value

ENPP1.1_protein 0.1872329 4.097407e-05

PNP.1_protein -0.2649669 4.669463e-09

$Pentose_and_glucuronate_interconversions_hum

estimate p.value

ALDH3A2.1_protein 0.2146441 2.406802e-06

$Pentose_phosphate_pathway_hum

estimate p.value

PGM2.1_protein -0.2487261 4.080762e-08

RPIA.1_protein -0.3820924 6.339777e-18

G6PD.1_protein 0.2748977 1.151886e-09

$Propanoate_metabolism_hum

estimate p.value

ACADM.1_protein -0.3079106 7.222179e-12

ACSS2.4_protein 0.2396906 1.278907e-07

LDHAL6B.1_protein -0.1893068 3.351193e-05

LDHB.1_protein -0.2690584 2.641359e-09

DBT.1_protein -0.2361817 1.968999e-07

BCKDHB.1_protein -0.1855124 4.833143e-05

ECHDC1.1_protein -0.1985847 1.327433e-05

$Purine_metabolism_hum

estimate p.value

PKLR.2_protein -0.2297486 4.268652e-07

PGM2.1_protein -0.2487261 4.080762e-08

ENPP1.1_protein 0.1872329 4.097407e-05

EEF2.2_protein -0.3212951 7.636219e-13

PAPSS2.1_protein 0.2237581 8.600234e-07

ATP9A.2_protein 0.2590142 1.051606e-08

ATP5F1.2_protein -0.2120829 3.188687e-06

ATP1A3.1_protein -0.1920371 2.563391e-05

ATP5A1.1_protein -0.2233905 8.972276e-07

ATP6V0C.1_protein 0.2324772 3.082780e-07

ATP2A1.1_protein -0.2028403 8.553621e-06

ATP8B1.1_protein 0.2325622 3.051506e-07

ATP5G2.1_protein -0.2267592 6.069407e-07

ATP2A3.1_protein -0.1962646 1.680251e-05

SPAST.2_protein -0.3038198 1.403444e-11

ATP6V0A2.1_protein -0.2414199 1.031341e-07

KATNA1.1_protein -0.3011376 2.157460e-11

ATP6V1G2.3_protein -0.2119544 3.233723e-06

ATP6AP1.1_protein 0.2899188 1.242801e-10

ATP6V1E1.1_protein 0.2227528 9.654782e-07

ATP11C.4_protein -0.2423170 9.218224e-08

ATP6V0E1.1_protein 0.3491871 4.875323e-15

ATP6V1D.1_protein 0.2007274 1.065172e-05

CFTR.2_protein 0.2007274 1.065172e-05

ADCY9.1_protein 0.2564976 1.473299e-08

ADCY6.1_protein 0.2522150 2.593750e-08

ADCY3.1_protein -0.1992049 1.245800e-05

TAF9.1_protein -0.2092772 4.322551e-06

AK2.5_protein -0.3150648 2.203927e-12

NUDT2.2_protein -0.3017731 1.949256e-11

DCK.2_protein -0.3173724 1.492611e-12

HPRT1.1_protein -0.2288188 4.764931e-07

PDE4A.1_protein -0.2162610 2.011595e-06

PDE3B.1_protein -0.1838414 5.665882e-05

CANT1.1_protein 0.1828582 6.217341e-05

ENTPD6.1_protein 0.2148188 2.360759e-06

TUFM.1_protein -0.1988612 1.290439e-05

EEF1A1.1_protein -0.2668023 3.620641e-09

POLD1.2_protein -0.3659864 1.803624e-16

POLE.1_protein -0.3247572 4.192202e-13

MYBBP1A.1_protein -0.2167226 1.910702e-06

POLG.1_protein -0.1900331 3.121776e-05

POLE2.1_protein -0.2682765 2.947383e-09

POLE3.2_protein -0.2836276 3.209718e-10

POLH.1_protein -0.2270100 5.893907e-07

POLQ.1_protein -0.2710715 1.988572e-09

POLA1.1_protein -0.3003830 2.432967e-11

POLR2A.1_protein -0.2384637 1.488318e-07

POLR1B.1_protein -0.3247723 4.181194e-13

PAPOLG.1_protein -0.3177794 1.392981e-12

PAPOLB.1_protein -0.1854330 4.869970e-05

POLR3A.2_protein -0.2037673 7.763119e-06

PAPOLA.1_protein -0.2037673 7.763119e-06

PNPT1.1_protein -0.1842153 5.468585e-05

NUDT5.1_protein -0.2046865 7.048383e-06

PPAT.1_protein -0.2843346 2.888479e-10

ATIC.1_protein -0.2213986 1.127324e-06

IMPDH2.1_protein -0.3133116 2.956683e-12

ADSSL1.1_protein 0.1858081 4.698411e-05

ADA.1_protein -0.1858134 4.696025e-05

PNP.1_protein -0.2649669 4.669463e-09

RRM2.1_protein -0.3082136 6.872530e-12

RRM1.1_protein -0.3202494 9.138838e-13

GART.3_protein -0.2890167 1.426012e-10

PAICS.1_protein -0.2853358 2.486511e-10

PFAS.1_protein -0.2966716 4.371606e-11

HUWE1.1_protein -0.1841220 5.517192e-05

HDDC3.1_protein -0.1999657 1.152173e-05

$Pyrimidine_metabolism_hum

estimate p.value

CAD.1_protein -0.2552310 1.743434e-08

DCK.2_protein -0.3173724 1.492611e-12

CANT1.1_protein 0.1828582 6.217341e-05

ENTPD6.1_protein 0.2148188 2.360759e-06

POLD1.2_protein -0.3659864 1.803624e-16

POLE.1_protein -0.3247572 4.192202e-13

MYBBP1A.1_protein -0.2167226 1.910702e-06

POLG.1_protein -0.1900331 3.121776e-05

POLE2.1_protein -0.2682765 2.947383e-09

POLE3.2_protein -0.2836276 3.209718e-10

POLH.1_protein -0.2270100 5.893907e-07

POLQ.1_protein -0.2710715 1.988572e-09

POLA1.1_protein -0.3003830 2.432967e-11

POLR2A.1_protein -0.2384637 1.488318e-07

POLR1B.1_protein -0.3247723 4.181194e-13

POLR3A.2_protein -0.2037673 7.763119e-06

PNPT1.1_protein -0.1842153 5.468585e-05

PNP.1_protein -0.2649669 4.669463e-09

RRM2.1_protein -0.3082136 6.872530e-12

RRM1.1_protein -0.3202494 9.138838e-13

ZCCHC11.1_protein -0.2234110 8.951201e-07

ZCCHC6.4_protein -0.2234110 8.951201e-07

UCK2.1_protein -0.2225542 9.877363e-07

TK2.1_protein 0.2054286 6.517503e-06

DHODH.1_protein -0.2442230 7.251566e-08

UPP1.1_protein 0.2896359 1.297637e-10

TXNRD2.5_protein -0.3884395 1.608669e-18

TXNRD1.1_protein -0.3751958 2.720577e-17

DUT.2_protein -0.3890502 1.407602e-18

TYMS.1_protein -0.2681073 3.018008e-09

$Pyruvate_metabolism_hum

estimate p.value

ACAT1.1_protein -0.2019688 9.366219e-06

ALDH1A3.1_protein 0.2120611 3.196291e-06

ALDH3B1.1_protein 0.2624636 6.585836e-09

ALDH3A2.1_protein 0.2146441 2.406802e-06

PKLR.2_protein -0.2297486 4.268652e-07

LDHAL6B.1_protein -0.1893068 3.351193e-05

LDHB.1_protein -0.2690584 2.641359e-09

LDHC.2_protein -0.2410739 1.076849e-07

ME2.1_protein -0.2786299 6.707190e-10

ME1.1_protein 0.2378140 1.612247e-07

ACYP1.1_protein -0.2863572 2.132744e-10

$Starch_and_sucrose_metabolism_hum

estimate p.value

PGM2.1_protein -0.2487261 4.080762e-08

UGDH.1_protein 0.2507144 3.154561e-08

GANC.1_protein -0.2472651 4.923646e-08

ENPP1.1_protein 0.1872329 4.097407e-05

UXS1.1_protein 0.2375562 1.664108e-07

AMY2B.1_protein -0.2154140 2.210145e-06

PYGB.1_protein 0.2809384 4.780642e-10

$Tyrosine_metabolism_hum

estimate p.value

ALDH1A3.1_protein 0.2120611 3.196291e-06

ALDH3B1.1_protein 0.2624636 6.585836e-09

MAOA.1_protein 0.2038570 7.690468e-06

TYRP1.1_protein 0.2379254 1.590304e-07

$Valine_leucine_and_isoleucine_biosynthesis_hum

estimate p.value

BCAT1.1_protein -0.2014008 9.934887e-06

SDSL.1_protein 0.2342948 2.476403e-07

$Valine_leucine_and_isoleucine_degradation_hum

estimate p.value

ACAT1.1_protein -0.2019688 9.366219e-06

HADH.1_protein -0.2723732 1.653031e-09

ALDH3A2.1_protein 0.2146441 2.406802e-06

ACADM.1_protein -0.3079106 7.222179e-12

OXCT1.1_protein -0.2768129 8.736265e-10

DBT.1_protein -0.2361817 1.968999e-07

HIBADH.1_protein 0.2123893 3.083730e-06

BCKDHB.1_protein -0.1855124 4.833143e-05

$Valine_leucine_isoleucine_degradation_hum

estimate p.value

BCAT1.1_protein -0.2014008 9.934887e-06

SDSL.1_protein 0.2342948 2.476403e-07

ACAT1.1_protein -0.2019688 9.366219e-06
