# Supplementary material for: Computational modeling of methionine cycle-based metabolism and DNA methylation and the implications for anti-cancer drug response prediction
Source: Oncotarget. 2018 Feb 21;9(32):22546–58. doi: 10.18632/oncotarget.24547 (PMC5989406; doi:10.18632/oncotarget.24547)
Supplement: Supplementary file 3 [file oncotarget-09-22546-s003.doc]

Supplementary Information 2: An input data format for AutoAnalyse

<CancerCells>

<Cell Age="unknown" Name="1321N1" Normalization="false" Sex="unknown" Survival="unknown">

<concentration name="GLA" object_ID="2906" value="376.498420"/>

<concentration name="PMM2" object_ID="4743" value="492.575137"/>

<concentration name="GUSB" object_ID="3087" value="1424.263139"/>

<concentration name="FAH" object_ID="2575" value="327.919981"/>

<concentration name="INPP5A" object_ID="3395" value="134.624763"/>

<concentration name="BCAT2" object_ID="1440" value="599.950640"/>

<concentration name="HDC" object_ID="3131" value="22.898799"/>

<concentration name="NME3" object_ID="17777" value="336.952168"/>

<concentration name="ITPKB" object_ID="3464" value="44.428083"/>

<concentration name="MBOAT2" object_ID="15272" value="662.510725"/>

<concentration name="HK2" object_ID="3176" value="144.427334"/>

<concentration name="CD38" object_ID="1743" value="30.347379"/>

<concentration name="AOX1" object_ID="1213" value="31.995564"/>

<concentration name="AGL" object_ID="1071" value="649.371384"/>

<concentration name="UGT2B4" object_ID="6242" value="8.928390"/>

<concentration name="ALDH3B1" object_ID="1129" value="70.282812"/>

<concentration name="B4GALT2" object_ID="1412" value="147.247568"/>

<concentration name="PLCG1" object_ID="4712" value="250.748976"/>

<concentration name="DNM1" object_ID="2310" value="35.336812"/>

<concentration name="PCCA" object_ID="4469" value="625.297960"/>

<concentration name="PDE5A" object_ID="4517" value="33.298840"/>

<concentration name="SHMT1" object_ID="5530" value="176.864415"/>

<concentration name="PNLIP" object_ID="4751" value="14.138920"/>

<concentration name="POLE4" object_ID="4771" value="266.040117"/>

<concentration name="PKLR" object_ID="4675" value="19.044467"/>

<concentration name="ADH6" object_ID="1047" value="15.842191"/>

<concentration name="AMPD1" object_ID="1173" value="13.917228"/>

<concentration name="ATP6V1E2" object_ID="1366" value="70.326670"/>

<concentration name="GCAT" object_ID="2858" value="250.922842"/>

<concentration name="NOS1" object_ID="4263" value="16.144826"/>

<concentration name="DGKI" object_ID="2257" value="25.228806"/>

<concentration name="NT5C3" object_ID="4332" value="793.837552"/>

<concentration name="TK1" object_ID="5971" value="1924.142590"/>

<concentration name="KATNAL1" object_ID="3506" value="121.659064"/>

<concentration name="MPI" object_ID="3995" value="109.728920"/>

<concentration name="NUDT9" object_ID="4354" value="455.655604"/>

<concentration name="PSPH" object_ID="5021" value="346.065185"/>

<concentration name="GRHPR" object_ID="3025" value="1113.588112"/>

<concentration name="CMAS" object_ID="11312" value="455.687189"/>

<concentration name="TYMS" object_ID="6179" value="10572.287922"/>

<concentration name="ATP10A" object_ID="10292" value="23.810071"/>

<concentration name="PDHA1" object_ID="4531" value="1348.369328"/>

<concentration name="SUCLG2" object_ID="5815" value="1013.338174"/>

<concentration name="IVD" object_ID="3469" value="184.707674"/>

<concentration name="ATIC" object_ID="1319" value="3453.874628"/>

<concentration name="PDE4D" object_ID="4515" value="18.562254"/>

<concentration name="DGUOK" object_ID="2261" value="1806.523116"/>

<concentration name="LCLAT1" object_ID="3634" value="1384.356693"/>

<concentration name="ENOSF1" object_ID="12482" value="40.126961"/>

<concentration name="MPST" object_ID="4000" value="297.243201"/>

<concentration name="GLYCTK" object_ID="2925" value="48.402176"/>

<concentration name="PIK3C2A" object_ID="4641" value="330.865258"/>

<concentration name="HADH" object_ID="3097" value="445.474796"/>

<concentration name="FAHD1" object_ID="7636" value="889.838679"/>

<concentration name="MTMR3" object_ID="7688" value="36.993503"/>

<concentration name="DNM3" object_ID="2313" value="23.305523"/>

<concentration name="AUH" object_ID="1384" value="327.511102"/>

<concentration name="DHDH" object_ID="2264" value="18.548106"/>

<concentration name="TAT" object_ID="5877" value="13.789517"/>

<concentration name="MCAT" object_ID="3861" value="457.967052"/>

<concentration name="ADCY7" object_ID="1039" value="58.113442"/>

<concentration name="IPPK" object_ID="3417" value="80.192755"/>

<concentration name="INPP5D" object_ID="3397" value="19.080142"/>

<concentration name="NADK" object_ID="4096" value="177.367762"/>

<concentration name="ATP6V1G2" object_ID="38940" value="34.456398"/>

<concentration name="ATP7A" object_ID="1372" value="101.849847"/>

<concentration name="ATP6V0E2" object_ID="17294" value="628.208651"/>

<concentration name="PIK3C3" object_ID="4644" value="240.851484"/>

<concentration name="GCK" object_ID="2862" value="20.554751"/>

<concentration name="L2HGDH" object_ID="3607" value="166.641047"/>

<concentration name="POLR1A" object_ID="4781" value="146.972253"/>

<concentration name="AADAT" object_ID="899" value="154.729021"/>

<concentration name="DLD" object_ID="2290" value="573.480253"/>

<concentration name="GALK2" object_ID="2809" value="105.741655"/>

<concentration name="PIP5KL1" object_ID="4667" value="1"/>

<concentration name="NANS" object_ID="4104" value="2668.845362"/>

<concentration name="NNT" object_ID="4257" value="340.615435"/>

<concentration name="TPO" object_ID="6084" value="12.686305"/>

<concentration name="MINPP1" object_ID="3931" value="1058.642674"/>

<concentration name="INPP1" object_ID="3392" value="432.423165"/>

<concentration name="OTC" object_ID="4406" value="13.924948"/>

<concentration name="SDHB" object_ID="5446" value="1902.921061"/>

<concentration name="PDE11A" object_ID="4507" value="12.128254"/>

<concentration name="DGKD" object_ID="2253" value="172.493717"/>

<concentration name="ACSL6" object_ID="988" value="11.122429"/>

<concentration name="ATP5J" object_ID="1346" value="7408.675861"/>

<concentration name="HDDC3" object_ID="13910" value="222.567634"/>

<concentration name="ALDH3B2" object_ID="1130" value="13.295812"/>

<concentration name="ATP6V1H" object_ID="1371" value="408.587144"/>

<concentration name="PGK2" object_ID="4576" value="23.698108"/>

<concentration name="GLS" object_ID="2918" value="67.037665"/>

<concentration name="CMPK2" object_ID="11695" value="18.287678"/>

<concentration name="DBH" object_ID="2187" value="18.124886"/>

<concentration name="AGK" object_ID="1070" value="339.578192"/>

<concentration name="ABHD5" object_ID="16873" value="101.307698"/>

<concentration name="PGK1" object_ID="4575" value="2942.853482"/>

<concentration name="CBS" object_ID="1707" value="113.181943"/>

<concentration name="NAPRT1" object_ID="13354" value="57.992724"/>

<concentration name="ITPA" object_ID="3461" value="810.573949"/>

<concentration name="IDH1" object_ID="3290" value="7383.043790"/>

<concentration name="GSS" object_ID="3050" value="630.171195"/>

<concentration name="INPPL1" object_ID="3402" value="94.313998"/>

<concentration name="LPL" object_ID="3699" value="18.426370"/>

<concentration name="ISYNA1" object_ID="3436" value="42.647817"/>

<concentration name="HADHA" object_ID="3098" value="1579.223885"/>

<concentration name="ATP8B3" object_ID="1376" value="12.139188"/>

<concentration name="ATP7B" object_ID="1373" value="40.636380"/>

<concentration name="ACAT1" object_ID="963" value="785.408826"/>

<concentration name="PNLIPRP3" object_ID="10483" value="10.444706"/>

<concentration name="POLR1B" object_ID="4782" value="759.602056"/>

<concentration name="ATP6V1A" object_ID="1359" value="462.528996"/>

<concentration name="DGKH" object_ID="2256" value="42.828522"/>

<concentration name="AFMID" object_ID="1065" value="47.971312"/>

<concentration name="ACO1" object_ID="970" value="730.023957"/>

<concentration name="APIP" object_ID="17343" value="1"/>

<concentration name="MDH2" object_ID="3875" value="6409.517316"/>

<concentration name="AKR1B1" object_ID="1106" value="7553.868742"/>

<concentration name="GFPT2" object_ID="2881" value="800.690012"/>

<concentration name="GLDC" object_ID="2910" value="254.496146"/>

<concentration name="MLYCD" object_ID="3958" value="60.585473"/>

<concentration name="NT5C1B" object_ID="4330" value="13.472073"/>

<concentration name="ACSBG2" object_ID="10328" value="21.785556"/>

<concentration name="ACSS2" object_ID="991" value="131.635062"/>

<concentration name="ECHS1" object_ID="2401" value="2778.325663"/>

<concentration name="NME6" object_ID="4251" value="306.660811"/>

<concentration name="ADCY8" object_ID="1040" value="15.345035"/>

<concentration name="ALDH3A1" object_ID="1127" value="18.740662"/>

<concentration name="SRR" object_ID="7482" value="167.080551"/>

<concentration name="HUWE1" object_ID="3274" value="49.587342"/>

<concentration name="ATP9A" object_ID="11999" value="1414.425035"/>

<concentration name="LDHAL6B" object_ID="3642" value="13.038446"/>

<concentration name="POLE2" object_ID="4769" value="891.814595"/>

<concentration name="ADCY6" object_ID="1038" value="100.496399"/>

<concentration name="POLRMT" object_ID="4806" value="1"/>

<concentration name="HSD17B10" object_ID="3240" value="1615.763130"/>

<concentration name="NPR2" object_ID="4279" value="49.340485"/>

<concentration name="ALAS1" object_ID="1117" value="598.704374"/>

<concentration name="CYB5RL" object_ID="48941" value="33.368155"/>

<concentration name="SARDH" object_ID="5415" value="12.535955"/>

<concentration name="LIPF" object_ID="16209" value="20.768138"/>

<concentration name="PPAT" object_ID="4840" value="293.842650"/>

<concentration name="OXCT1" object_ID="4411" value="469.994479"/>

<concentration name="AHCYL1" object_ID="9266" value="1838.100584"/>

<concentration name="PSAT1" object_ID="5005" value="5227.842637"/>

<concentration name="SYNJ2" object_ID="5847" value="79.467883"/>

<concentration name="PLCB1" object_ID="4704" value="87.809188"/>

<concentration name="PIK3C2G" object_ID="4643" value="15.171586"/>

<concentration name="ALDH1B1" object_ID="1124" value="118.940810"/>

<concentration name="ACOX1" object_ID="975" value="225.471301"/>

<concentration name="CFTR" object_ID="16915" value="14.302545"/>

<concentration name="CYP4A22" object_ID="2166" value="18.864474"/>

<concentration name="ATP5D" object_ID="1338" value="215.120321"/>

<concentration name="POLS" object_ID="4807" value="426.617696"/>

<concentration name="LPCAT3" object_ID="8946" value="39.325685"/>

<concentration name="PFKP" object_ID="4562" value="2181.341601"/>

<concentration name="ATP2B2" object_ID="6949" value="14.483115"/>

<concentration name="ACADSB" object_ID="959" value="109.728920"/>

<concentration name="PGAM1" object_ID="4565" value="1"/>

<concentration name="PRHOXNB" object_ID="54648" value="1"/>

<concentration name="ATP6V1C2" object_ID="1363" value="15.397241"/>

<concentration name="AMDHD2" object_ID="17247" value="12.961852"/>

<concentration name="ATP1A2" object_ID="1329" value="14.512257"/>

<concentration name="DUT" object_ID="2378" value="1048.419226"/>

<concentration name="ACSL3" object_ID="985" value="1781.652178"/>

<concentration name="PC" object_ID="4468" value="69.199994"/>

<concentration name="MAT1A" object_ID="3849" value="18.256015"/>

<concentration name="ALDH6A1" object_ID="1133" value="317.871530"/>

<concentration name="ATP6V1B2" object_ID="1361" value="616.903063"/>

<concentration name="ABHD6" object_ID="12429" value="135.673956"/>

<concentration name="GNPNAT1" object_ID="2967" value="760.497661"/>

<concentration name="XDH" object_ID="6381" value="66.408684"/>

<concentration name="ACSL5" object_ID="987" value="31.122801"/>

<concentration name="NSF" object_ID="4325" value="1084.639575"/>

<concentration name="MCCC1" object_ID="3862" value="392.214755"/>

<concentration name="GK" object_ID="2902" value="65.927113"/>

<concentration name="PRPS1" object_ID="4991" value="531.785268"/>

<concentration name="ATP4A" object_ID="1333" value="14.140880"/>

<concentration name="AGXT" object_ID="1079" value="17.289209"/>

<concentration name="PAPSS1" object_ID="4448" value="1180.350819"/>

<concentration name="IPMK" object_ID="3410" value="29.768198"/>

<concentration name="ATP6V0D1" object_ID="1356" value="1611.289488"/>

<concentration name="HADHB" object_ID="3099" value="4408.279551"/>

<concentration name="RPE" object_ID="5358" value="1470.407179"/>

<concentration name="PDE7B" object_ID="4522" value="36.421035"/>

<concentration name="DNMT3B" object_ID="2316" value="191.526472"/>

<concentration name="MCCC2" object_ID="3863" value="194.874296"/>

<concentration name="PPAP2B" object_ID="4829" value="800.690012"/>

<concentration name="ATP6V1G1" object_ID="1368" value="185.799138"/>

<concentration name="PNP" object_ID="49946" value="970.577765"/>

<concentration name="ADPGK" object_ID="16573" value="184.439008"/>

<concentration name="IMPA1" object_ID="3374" value="1810.283584"/>

<concentration name="EEF1A2" object_ID="2412" value="34.308639"/>

<concentration name="FBP2" object_ID="2630" value="24.506627"/>

<concentration name="PDE4C" object_ID="4514" value="18.283876"/>

<concentration name="UAP1L1" object_ID="17223" value="75.557340"/>

<concentration name="TSTA3" object_ID="6151" value="507.794163"/>

<concentration name="AACS" object_ID="897" value="223.681170"/>

<concentration name="ATP6V1E1" object_ID="1365" value="1369.088507"/>

<concentration name="MOGAT3" object_ID="14691" value="15.946855"/>

<concentration name="ADCY5" object_ID="1037" value="22.190957"/>

<concentration name="REV3L" object_ID="5270" value="1006.199038"/>

<concentration name="ACSM5" object_ID="7838" value="14.298580"/>

<concentration name="NT5M" object_ID="4334" value="34.320532"/>

<concentration name="TXNRD1" object_ID="6175" value="1210.175024"/>

<concentration name="DONSON" object_ID="8854" value="1316.051290"/>

<concentration name="DDC" object_ID="2209" value="17.223423"/>

<concentration name="SHMT2" object_ID="5531" value="1264.192109"/>

<concentration name="PDE8A" object_ID="4523" value="378.775708"/>

<concentration name="DCXR" object_ID="2206" value="1070.448743"/>

<concentration name="ALDH2" object_ID="1126" value="38.088934"/>

<concentration name="CAD" object_ID="1654" value="1252.851754"/>

<concentration name="AGXT2" object_ID="1080" value="23.320067"/>

<concentration name="DAK" object_ID="2178" value="42.282829"/>

<concentration name="ATP2B4" object_ID="14701" value="356.535025"/>

<concentration name="INPP5E" object_ID="3398" value="166.606399"/>

<concentration name="TALDO1" object_ID="5868" value="3158.447770"/>

<concentration name="TMEM91" object_ID="14343" value="32.466951"/>

<concentration name="UGT2B17" object_ID="6240" value="11.870428"/>

<concentration name="ATP8B1" object_ID="1375" value="84.706455"/>

<concentration name="PGLS" object_ID="4577" value="873.582412"/>

<concentration name="ALDH5A1" object_ID="1132" value="111.577320"/>

<concentration name="NAGK" object_ID="4099" value="1451.169174"/>

<concentration name="RGN" object_ID="5289" value="13.404077"/>

<concentration name="PFAS" object_ID="4554" value="1042.621645"/>

<concentration name="AMDHD1" object_ID="1169" value="50.258650"/>

<concentration name="ASS1" object_ID="1312" value="4320.552211"/>

<concentration name="PDE3B" object_ID="4511" value="20.360485"/>

<concentration name="DNM1L" object_ID="2311" value="2078.028895"/>

<concentration name="GUCY1A3" object_ID="3081" value="16.292092"/>

<concentration name="PPAP2C" object_ID="4830" value="15.886176"/>

<concentration name="NUDT14" object_ID="6996" value="221.045568"/>

<concentration name="MGLL" object_ID="3918" value="560.239541"/>

<concentration name="AK2" object_ID="1092" value="363.094181"/>

<concentration name="C1orf57" object_ID="13718" value="949.615715"/>

<concentration name="C17orf48" object_ID="18005" value="166.283361"/>

<concentration name="ACSM3" object_ID="14100" value="31.847319"/>

<concentration name="GNE" object_ID="2952" value="512.461567"/>

<concentration name="DHDPSL" object_ID="18159" value="35.479161"/>

<concentration name="ITPKA" object_ID="3463" value="48.627483"/>

<concentration name="FOLH1B" object_ID="12510" value="1"/>

<concentration name="DHX58" object_ID="2276" value="23.090060"/>

<concentration name="PDE6C" object_ID="8098" value="9.635102"/>

<concentration name="ASPA" object_ID="1307" value="13.201226"/>

<concentration name="PKM2" object_ID="4676" value="4258.117366"/>

<concentration name="POLK" object_ID="4776" value="274.773679"/>

<concentration name="ATP6V1G3" object_ID="1370" value="18.487779"/>

<concentration name="HAL" object_ID="3102" value="16.602171"/>

<concentration name="ADSS" object_ID="1058" value="855.722966"/>

<concentration name="IMPDH1" object_ID="3377" value="1126.006937"/>

<concentration name="ABP1" object_ID="948" value="12.209227"/>

<concentration name="ADA" object_ID="1007" value="616.347428"/>

<concentration name="NAALADL1" object_ID="14617" value="23.284532"/>

<concentration name="NPL" object_ID="7041" value="52.556598"/>

<concentration name="FH" object_ID="2690" value="814.629359"/>

<concentration name="ARG2" object_ID="1256" value="147.615458"/>

<concentration name="PCCB" object_ID="4470" value="1264.192109"/>

<concentration name="DBT" object_ID="2189" value="144.007486"/>

<concentration name="UGT2B11" object_ID="6238" value="1"/>

<concentration name="MTAP" object_ID="4027" value="17.529343"/>

<concentration name="ACSL1" object_ID="984" value="277.299268"/>

<concentration name="PNLIPRP1" object_ID="4752" value="20.250702"/>

<concentration name="DDO" object_ID="2213" value="18.800511"/>

<concentration name="PAPOLB" object_ID="10375" value="12.773660"/>

<concentration name="PAPSS2" object_ID="4449" value="162.534255"/>

<concentration name="PLCB4" object_ID="4707" value="223.572665"/>

<concentration name="UGT2A3" object_ID="6236" value="11.951336"/>

<concentration name="ABHD12" object_ID="939" value="334.601476"/>

<concentration name="ACY3" object_ID="1004" value="23.452991"/>

<concentration name="NT5E" object_ID="4333" value="925.442792"/>

<concentration name="MTR" object_ID="4040" value="676.712862"/>

<concentration name="ATP6V0C" object_ID="1355" value="2967.433533"/>

<concentration name="UROC1" object_ID="6268" value="13.785694"/>

<concentration name="H6PD" object_ID="3095" value="61.261127"/>

<concentration name="GMPR2" object_ID="2931" value="1071.190978"/>

<concentration name="PDE4A" object_ID="4512" value="15.784099"/>

<concentration name="UCK2" object_ID="6216" value="364.026584"/>

<concentration name="ATP5H" object_ID="1344" value="1"/>

<concentration name="ENTPD2" object_ID="2482" value="17.254491"/>

<concentration name="C12orf5" object_ID="11679" value="1255.459697"/>

<concentration name="PPAP2A" object_ID="4828" value="575.112347"/>

<concentration name="HAGHL" object_ID="3101" value="86.432381"/>

<concentration name="ATP6V0A2" object_ID="1352" value="107.254919"/>

<concentration name="DGKQ" object_ID="2259" value="57.222079"/>

<concentration name="ALDH3A2" object_ID="1128" value="622.875493"/>

<concentration name="ATP10D" object_ID="16524" value="548.709978"/>

<concentration name="DLAT" object_ID="2288" value="773.737292"/>

<concentration name="MAOA" object_ID="3778" value="57.492426"/>

<concentration name="HEXA" object_ID="3147" value="534.408817"/>

<concentration name="KATNA1" object_ID="14547" value="540.031593"/>

<concentration name="GUCY1B3" object_ID="3082" value="114.929074"/>

<concentration name="PGD" object_ID="4572" value="2060.816023"/>

<concentration name="AMD1" object_ID="1168" value="1824.139060"/>

<concentration name="DCT" object_ID="2202" value="19.916607"/>

<concentration name="ADH5" object_ID="1046" value="1743.777193"/>

<concentration name="GALM" object_ID="2810" value="236.943619"/>

<concentration name="GLB1" object_ID="2907" value="1233.891651"/>

<concentration name="PBEF1" object_ID="4102" value="297.985847"/>

<concentration name="PDE6B" object_ID="14147" value="29.537995"/>

<concentration name="ACADL" object_ID="956" value="13.159201"/>

<concentration name="DGAT2" object_ID="2246" value="187.662945"/>

<concentration name="PRPS1L1" object_ID="4992" value="13.492633"/>

<concentration name="ATP5B" object_ID="1336" value="4860.873923"/>

<concentration name="DNMT1" object_ID="2314" value="3583.118397"/>

<concentration name="TYRP1" object_ID="6183" value="17.334809"/>

<concentration name="ACOT12" object_ID="973" value="11.374254"/>

<concentration name="COMT" object_ID="1994" value="282.420304"/>

<concentration name="ACOX3" object_ID="976" value="168.382687"/>

<concentration name="GAD2" object_ID="2800" value="16.099009"/>

<concentration name="ACYP2" object_ID="1006" value="361.411850"/>

<concentration name="GAPDH" object_ID="2832" value="29044.955519"/>

<concentration name="ACAA2" object_ID="951" value="61.380139"/>

<concentration name="UGT2B10" object_ID="6237" value="1"/>

<concentration name="ADI1" object_ID="18074" value="1299.733387"/>

<concentration name="TXNRD3" object_ID="47947" value="114.444157"/>

<concentration name="GNMT" object_ID="2963" value="15.042787"/>

<concentration name="DNM2" object_ID="2312" value="63.880336"/>

<concentration name="DMGDH" object_ID="2300" value="11.273003"/>

<concentration name="PNMT" object_ID="4755" value="43.871131"/>

<concentration name="ADCY1" object_ID="1032" value="16.315825"/>

<concentration name="UGT1A6" object_ID="6231" value="12.136664"/>

<concentration name="MAT2A" object_ID="18599" value="690.261879"/>

<concentration name="MOGAT2" object_ID="15863" value="18.821373"/>

<concentration name="GLUL" object_ID="2924" value="108.496147"/>

<concentration name="ALAS2" object_ID="1118" value="17.974752"/>

<concentration name="GALE" object_ID="2807" value="1168.142045"/>

<concentration name="PAICS" object_ID="4431" value="3331.601196"/>

<concentration name="ALLC" object_ID="1155" value="15.896090"/>

<concentration name="B4GALT1" object_ID="1411" value="38.009812"/>

<concentration name="GDA" object_ID="2870" value="15.473203"/>

<concentration name="AGPAT3" object_ID="1075" value="149.003251"/>

<concentration name="DNMT3A" object_ID="2315" value="44.249830"/>

<concentration name="ACSL4" object_ID="986" value="1"/>

<concentration name="NT5C" object_ID="4328" value="268.336593"/>

<concentration name="MCEE" object_ID="3864" value="326.762814"/>

<concentration name="ABHD14A" object_ID="9822" value="1"/>

<concentration name="GCDH" object_ID="2859" value="289.556285"/>

<concentration name="GUCY2F" object_ID="3085" value="14.779238"/>

<concentration name="AK1" object_ID="1091" value="899.949352"/>

<concentration name="ATP5E" object_ID="1339" value="1"/>

<concentration name="AGPAT6" object_ID="1077" value="83.332075"/>

<concentration name="IMPA2" object_ID="3375" value="1006.757148"/>

<concentration name="FBP1" object_ID="2629" value="18.049663"/>

<concentration name="ATP6V0A4" object_ID="1353" value="14.493157"/>

<concentration name="AHCY" object_ID="1082" value="1760.781284"/>

<concentration name="AK3L1" object_ID="1094" value="1094.457335"/>

<concentration name="GUCY1A2" object_ID="3080" value="27.886597"/>

<concentration name="CRYL1" object_ID="2055" value="38.044078"/>

<concentration name="DCI" object_ID="2193" value="745.570000"/>

<concentration name="NUDT16" object_ID="18083" value="67.223791"/>

<concentration name="BDH1" object_ID="1459" value="31.701957"/>

<concentration name="PIP4K2C" object_ID="4662" value="443.810503"/>

<concentration name="ATP11B" object_ID="17186" value="250.262791"/>

<concentration name="UPP2" object_ID="6258" value="12.068714"/>

<concentration name="NUDT12" object_ID="4350" value="221.014927"/>

<concentration name="EEF2" object_ID="10256" value="11409.911650"/>

<concentration name="ECHDC1" object_ID="11837" value="2007.243603"/>

<concentration name="INPP4B" object_ID="3394" value="743.505704"/>

<concentration name="ACYP1" object_ID="1005" value="464.295665"/>

<concentration name="NUDT2" object_ID="4351" value="295.333254"/>

<concentration name="GYS2" object_ID="10592" value="12.992436"/>

<concentration name="NME4" object_ID="4250" value="1904.240522"/>

<concentration name="LDHAL6A" object_ID="3641" value="13.958771"/>

<concentration name="SI" object_ID="5536" value="11.427200"/>

<concentration name="NME1-NME2" object_ID="36764" value="1"/>

<concentration name="ATP5A1" object_ID="1335" value="9261.306406"/>

<concentration name="GMPR" object_ID="2930" value="27.923348"/>

<concentration name="AICDA" object_ID="1088" value="11.485168"/>

<concentration name="C9orf41" object_ID="18395" value="59.207170"/>

<concentration name="HPRT1" object_ID="3221" value="2440.565913"/>

<concentration name="RRM1" object_ID="5386" value="2296.148128"/>

<concentration name="PCK1" object_ID="4485" value="14.107593"/>

<concentration name="HGD" object_ID="3152" value="15.494669"/>

<concentration name="ENO2" object_ID="2471" value="420.219704"/>

<concentration name="ACSM1" object_ID="989" value="11.074733"/>

<concentration name="HMGCL" object_ID="3186" value="437.548764"/>

<concentration name="GPT2" object_ID="3011" value="406.778608"/>

<concentration name="NADSYN1" object_ID="4097" value="71.184933"/>

<concentration name="RRM2" object_ID="5387" value="5304.496123"/>

<concentration name="AOC3" object_ID="1211" value="10.253183"/>

<concentration name="GAD1" object_ID="2799" value="47.209327"/>

<concentration name="HMGCS1" object_ID="3188" value="502.263282"/>

<concentration name="NAT8L" object_ID="37559" value="42.855249"/>

<concentration name="ATP1A3" object_ID="1330" value="25.326925"/>

<concentration name="UGP2" object_ID="6225" value="3220.346029"/>

<concentration name="CEL" object_ID="1833" value="19.641048"/>

<concentration name="GNPDA1" object_ID="2965" value="1286.289798"/>

<concentration name="UPB1" object_ID="6254" value="21.491585"/>

<concentration name="HNMT" object_ID="3196" value="34.556855"/>

<concentration name="MAT2B" object_ID="3850" value="1863.759512"/>

<concentration name="DERA" object_ID="2239" value="1467.352730"/>

<concentration name="PDE3A" object_ID="16907" value="28.746213"/>

<concentration name="ATP6V0B" object_ID="1354" value="1489.900220"/>

<concentration name="CAT" object_ID="1699" value="1259.818337"/>

<concentration name="ACSS3" object_ID="992" value="230.145113"/>

<concentration name="PIK3CG" object_ID="4647" value="11.618887"/>

<concentration name="FUK" object_ID="2759" value="71.229354"/>

<concentration name="ATP5G3" object_ID="1343" value="18.349896"/>

<concentration name="LIPG" object_ID="12147" value="11.479596"/>

<concentration name="ADSSL1" object_ID="1059" value="60.179491"/>

<concentration name="CNDP2" object_ID="13458" value="67.410434"/>

<concentration name="AGPAT2" object_ID="1074" value="171.527966"/>

<concentration name="OCRL" object_ID="4379" value="140.663090"/>

<concentration name="AKR1A1" object_ID="1105" value="1593.518442"/>

<concentration name="PGAM2" object_ID="4566" value="12.192314"/>

<concentration name="G6PC" object_ID="2784" value="14.094887"/>

<concentration name="GMPS" object_ID="2932" value="1"/>

<concentration name="PGM2L1" object_ID="18695" value="100.273738"/>

<concentration name="CDO1" object_ID="1819" value="15.082462"/>

<concentration name="ACSF3" object_ID="16835" value="27.929155"/>

<concentration name="ADCY3" object_ID="1035" value="243.757027"/>

<concentration name="TYR" object_ID="6180" value="14.826437"/>

<concentration name="AK3" object_ID="1093" value="1152.059888"/>

<concentration name="DHODH" object_ID="2266" value="76.532467"/>

<concentration name="BCKDHA" object_ID="1443" value="206.743763"/>

<concentration name="ENPP1" object_ID="2474" value="28.324920"/>

<concentration name="NAALAD2" object_ID="16527" value="11.678637"/>

<concentration name="BCAT1" object_ID="1439" value="959.673603"/>

<concentration name="ATPGD1" object_ID="13169" value="25.096252"/>

<concentration name="NIT2" object_ID="14026" value="1754.689443"/>

<concentration name="PMM1" object_ID="4742" value="266.483057"/>

<concentration name="CTPS" object_ID="2106" value="1156.861148"/>

<concentration name="AGPAT1" object_ID="25244" value="213.663994"/>

<concentration name="POLR2A" object_ID="4786" value="30.592371"/>

<concentration name="LDHA" object_ID="3640" value="1"/>

<concentration name="DNMT3L" object_ID="13145" value="25.455403"/>

<concentration name="ADH1A" object_ID="1042" value="11.987841"/>

<concentration name="CPT1B" object_ID="2037" value="1"/>

<concentration name="DCTPP1" object_ID="19014" value="3038.197625"/>

<concentration name="SORD" object_ID="5667" value="1"/>

<concentration name="ENOPH1" object_ID="16949" value="2405.298117"/>

<concentration name="ATP8B2" object_ID="13300" value="241.939078"/>

<concentration name="GNPDA2" object_ID="2966" value="409.636369"/>

<concentration name="TYMP" object_ID="6178" value="30.798752"/>

<concentration name="NMNAT3" object_ID="4255" value="20.807042"/>

<concentration name="TKT" object_ID="5973" value="1446.148510"/>

<concentration name="PDE1C" object_ID="4509" value="20.422676"/>

<concentration name="DAO" object_ID="2179" value="19.065600"/>

<concentration name="PIP5K2A" object_ID="4660" value="345.945269"/>

<concentration name="POLQ" object_ID="4780" value="636.051600"/>

<concentration name="PDE7A" object_ID="4521" value="180.868831"/>

<concentration name="GFM1" object_ID="13628" value="1054.980049"/>

<concentration name="ASNA1" object_ID="15166" value="2932.671982"/>

<concentration name="OGDHL" object_ID="4383" value="30.372631"/>

<concentration name="OXCT2" object_ID="4412" value="1"/>

<concentration name="PGM2" object_ID="4580" value="348.762230"/>

<concentration name="AGPAT9" object_ID="11087" value="305.684587"/>

<concentration name="MDH1" object_ID="3874" value="7231.102946"/>

<concentration name="PLCD4" object_ID="4710" value="54.244313"/>

<concentration name="DCTD" object_ID="2203" value="677.839547"/>

<concentration name="INPP4A" object_ID="3393" value="50.029253"/>

<concentration name="PIK3CB" object_ID="4645" value="189.795240"/>

<concentration name="MTMR1" object_ID="4036" value="143.638631"/>

<concentration name="LDHB" object_ID="3643" value="16406.728798"/>

<concentration name="ASL" object_ID="1304" value="326.717518"/>

<concentration name="ENTPD5" object_ID="2484" value="58.234411"/>

<concentration name="DGKA" object_ID="2251" value="106.292795"/>

<concentration name="GUCY2C" object_ID="3083" value="11.569062"/>

<concentration name="ATP6V0E1" object_ID="1358" value="6063.772869"/>

<concentration name="GUK1" object_ID="3086" value="1663.492908"/>

<concentration name="POLG" object_ID="4772" value="112.034558"/>

<concentration name="TRDMT1" object_ID="10989" value="127.442265"/>

<concentration name="SLC2A11" object_ID="7357" value="22.160215"/>

<concentration name="NME5" object_ID="16281" value="16.014424"/>

<concentration name="PDE1A" object_ID="4508" value="119.768109"/>

<concentration name="NT5C1A" object_ID="4329" value="17.623151"/>

<concentration name="PTEN" object_ID="496" value="41.689148"/>

<concentration name="XYLB" object_ID="6399" value="26.704297"/>

<concentration name="ACLY" object_ID="968" value="1160.877479"/>

<concentration name="RRM2B" object_ID="5388" value="260.926561"/>

<concentration name="PGP" object_ID="55246" value="175.581861"/>

<concentration name="ATP4B" object_ID="1334" value="12.975336"/>

<concentration name="TPI1" object_ID="6080" value="5832.909601"/>

<concentration name="PFKFB3" object_ID="4558" value="527.709447"/>

<concentration name="MIOX" object_ID="3932" value="12.802911"/>

<concentration name="ALDOA" object_ID="1136" value="9014.301568"/>

<concentration name="ATP2C1" object_ID="8487" value="399.513377"/>

<concentration name="ATP5G1" object_ID="1341" value="796.538343"/>

<concentration name="POLR2B" object_ID="4787" value="2449.038944"/>

<concentration name="PIK3CD" object_ID="4646" value="29.109134"/>

<concentration name="DTYMK" object_ID="2352" value="3106.339933"/>

<concentration name="PIP5K1B" object_ID="4664" value="11.200568"/>

<concentration name="PGAM4" object_ID="4567" value="1"/>

<concentration name="ME2" object_ID="3880" value="806.091612"/>

<concentration name="HPD" object_ID="3218" value="16.564239"/>

<concentration name="ARG1" object_ID="1255" value="13.281995"/>

<concentration name="IMPDH2" object_ID="3378" value="6784.374058"/>

<concentration name="NMNAT2" object_ID="4254" value="66.335075"/>

<concentration name="NNMT" object_ID="4256" value="1400.765751"/>

<concentration name="GLS2" object_ID="2919" value="14.463051"/>

<concentration name="G6PD" object_ID="2786" value="595.930386"/>

<concentration name="HIBADH" object_ID="3158" value="262.650405"/>

<concentration name="MTM1" object_ID="11550" value="23.232943"/>

<concentration name="ACADS" object_ID="958" value="28.802058"/>

<concentration name="ADH7" object_ID="1048" value="12.791380"/>

<concentration name="POLI" object_ID="4775" value="84.016454"/>

<concentration name="GSTZ1" object_ID="3066" value="166.018476"/>

<concentration name="GOT2" object_ID="2975" value="1352.112989"/>

<concentration name="ACAA1" object_ID="950" value="410.745227"/>

<concentration name="HKDC1" object_ID="8671" value="31.966746"/>

<concentration name="INPP5K" object_ID="3401" value="138.361620"/>

<concentration name="ENTPD3" object_ID="569" value="23.660358"/>

<concentration name="DPYS" object_ID="2333" value="13.170151"/>

<concentration name="TCIRG1" object_ID="5900" value="204.917587"/>

<concentration name="PIPOX" object_ID="4668" value="13.939433"/>

<concentration name="DGKE" object_ID="2254" value="13.168326"/>

<concentration name="GK2" object_ID="16385" value="16.278547"/>

<concentration name="HK3" object_ID="3177" value="26.576883"/>

<concentration name="TNNI3K" object_ID="2737" value="1"/>

<concentration name="DGKB" object_ID="2252" value="11.700514"/>

<concentration name="GPAM" object_ID="2978" value="220.968973"/>

<concentration name="CS" object_ID="2057" value="1"/>

<concentration name="PFKFB1" object_ID="4556" value="18.137453"/>

<concentration name="ME1" object_ID="3879" value="876.797576"/>

<concentration name="AOC2" object_ID="1210" value="33.052787"/>

<concentration name="PIK3CA" object_ID="316" value="445.289567"/>

<concentration name="GLO1" object_ID="2915" value="4966.452507"/>

<concentration name="PFKFB2" object_ID="4557" value="29.070823"/>

<concentration name="RPIA" object_ID="5361" value="578.470669"/>

<concentration name="NAGS" object_ID="4100" value="44.858205"/>

<concentration name="PIP5K2B" object_ID="4661" value="82.441566"/>

<concentration name="UGDH" object_ID="6224" value="502.263282"/>

<concentration name="SLC27A2" object_ID="15472" value="19.270205"/>

<concentration name="SDS" object_ID="5449" value="24.605348"/>

<concentration name="MOGAT1" object_ID="17063" value="20.332279"/>

<concentration name="PIK3C2B" object_ID="4642" value="316.881582"/>

<concentration name="PECI" object_ID="4546" value="1928.147895"/>

<concentration name="ATP9B" object_ID="11244" value="33.642197"/>

<concentration name="INPP5J" object_ID="3400" value="38.481683"/>

<concentration name="C9orf103" object_ID="13465" value="95.504711"/>

<concentration name="GANC" object_ID="2831" value="34.886573"/>

<concentration name="ATP2B1" object_ID="14900" value="488.258043"/>

<concentration name="ATP2A3" object_ID="9222" value="20.021803"/>

<concentration name="ENPP3" object_ID="2476" value="14.235290"/>

<concentration name="ITPK1" object_ID="3462" value="32.171248"/>

<concentration name="GBA3" object_ID="2850" value="15.647931"/>

<concentration name="GCLC" object_ID="2863" value="245.946378"/>

<concentration name="TST" object_ID="11416" value="925.891929"/>

<concentration name="GUCY2D" object_ID="3084" value="22.716993"/>

<concentration name="SUCLG1" object_ID="5814" value="4237.506894"/>

<concentration name="AMY1A" object_ID="8927" value="1"/>

<concentration name="SDHA" object_ID="5445" value="1"/>

<concentration name="CPT2" object_ID="2039" value="255.202738"/>

<concentration name="PTCD1" object_ID="5026" value="1"/>

<concentration name="PHGDH" object_ID="4597" value="1544.581199"/>

<concentration name="NME1" object_ID="4248" value="11529.161731"/>

<concentration name="CANT1" object_ID="1676" value="426.528993"/>

<concentration name="CMPK1" object_ID="1953" value="1785.360875"/>

<concentration name="GPT" object_ID="3010" value="21.764425"/>

<concentration name="ACSM2" object_ID="13126" value="1"/>

<concentration name="PIKFYVE" object_ID="4666" value="132.110379"/>

<concentration name="CPS1" object_ID="2034" value="239.237544"/>

<concentration name="SUCLA2" object_ID="5813" value="772.290599"/>

<concentration name="PDE6A" object_ID="12990" value="14.180141"/>

<concentration name="GART" object_ID="2836" value="1046.966815"/>

<concentration name="UGT2B28" object_ID="6241" value="1"/>

<concentration name="ADH4" object_ID="1045" value="12.272864"/>

<concentration name="ENO3" object_ID="2472" value="54.112875"/>

<concentration name="CNDP1" object_ID="1957" value="23.604663"/>

<concentration name="GFM2" object_ID="14649" value="354.661766"/>

<concentration name="FPGT" object_ID="187458" value="56.917485"/>

<concentration name="UGT2B15" object_ID="6239" value="13.627037"/>

<concentration name="POLR3A" object_ID="4797" value="134.252021"/>

<concentration name="SPAST" object_ID="7718" value="225.705850"/>

<concentration name="TKTL2" object_ID="5975" value="16.347521"/>

<concentration name="UGT2B7" object_ID="6243" value="1"/>

<concentration name="UMPS" object_ID="6251" value="88.530336"/>

<concentration name="HAO1" object_ID="3104" value="15.553852"/>

<concentration name="TAF9" object_ID="5865" value="2055.110141"/>

<concentration name="PLCG2" object_ID="4713" value="32.113322"/>

<concentration name="UCKL1" object_ID="14259" value="178.440588"/>

<concentration name="FHIT" object_ID="2691" value="30.414766"/>

<concentration name="INPP5B" object_ID="3396" value="132.220311"/>

<concentration name="VILL" object_ID="8192" value="18.962799"/>

<concentration name="NME7" object_ID="4252" value="1525.429722"/>

<concentration name="PNPT1" object_ID="4758" value="1601.269029"/>

<concentration name="ATP12A" object_ID="1325" value="30.309539"/>

<concentration name="TH" object_ID="5949" value="16.618290"/>

<concentration name="UXS1" object_ID="6289" value="632.139870"/>

<concentration name="LALBA" object_ID="3608" value="29.630273"/>

<concentration name="TNRC6B" object_ID="12615" value="164.164685"/>

<concentration name="ADH1C" object_ID="1044" value="12.376230"/>

<concentration name="HAGH" object_ID="3100" value="358.791027"/>

<concentration name="POLE" object_ID="4768" value="144.127318"/>

<concentration name="ATP5G2" object_ID="1342" value="5282.481155"/>

<concentration name="PFKM" object_ID="4561" value="948.760410"/>

<concentration name="SRM" object_ID="5728" value="1436.159233"/>

<concentration name="ALDH1A3" object_ID="1123" value="28.273920"/>

<concentration name="ATP5L" object_ID="1348" value="1878.024280"/>

<concentration name="GCKR" object_ID="18377" value="16.222227"/>

<concentration name="BHMT2" object_ID="1471" value="14.479100"/>

<concentration name="SYNJ1" object_ID="5846" value="89.461807"/>

<concentration name="POLD1" object_ID="4763" value="247.845972"/>

<concentration name="GALT" object_ID="2828" value="80.991579"/>

<concentration name="PRUNE" object_ID="5003" value="134.624763"/>

<concentration name="ALDH4A1" object_ID="1131" value="47.419219"/>

<concentration name="BST1" object_ID="1511" value="12.224470"/>

<concentration name="ATP6V0A1" object_ID="1351" value="96.476121"/>

<concentration name="GALK1" object_ID="2808" value="47.944719"/>

<concentration name="ATP5C1" object_ID="1337" value="6191.185626"/>

<concentration name="PFKL" object_ID="4560" value="154.065497"/>

<concentration name="POLA1" object_ID="4760" value="754.041439"/>

<concentration name="ACSM4" object_ID="18419" value="1"/>

<concentration name="UPP1" object_ID="6257" value="106.123474"/>

<concentration name="CHIA" object_ID="1878" value="24.462501"/>

<concentration name="ACO2" object_ID="971" value="578.069843"/>

<concentration name="IDH3A" object_ID="3292" value="843.941975"/>

<concentration name="FTCD" object_ID="2753" value="20.927093"/>

<concentration name="NUDT5" object_ID="4352" value="4797.276902"/>

<concentration name="CTPS2" object_ID="2107" value="194.644801"/>

<concentration name="MUT" object_ID="688" value="384.382808"/>

<concentration name="GFPT1" object_ID="2880" value="619.731719"/>

<concentration name="AMPD3" object_ID="1175" value="33.586278"/>

<concentration name="ALDOC" object_ID="1138" value="36.885963"/>

<concentration name="TKTL1" object_ID="5974" value="15.216873"/>

<concentration name="PGM1" object_ID="4579" value="1602.379329"/>

<concentration name="ENTPD4" object_ID="2483" value="151.429240"/>

<concentration name="MTMR2" object_ID="4037" value="392.541126"/>

<concentration name="CPT1A" object_ID="2036" value="81.064592"/>

<concentration name="CPT1C" object_ID="2038" value="106.403367"/>

<concentration name="DCK" object_ID="2194" value="742.784551"/>

<concentration name="ABAT" object_ID="907" value="17.978491"/>

<concentration name="GLUD2" object_ID="2923" value="81.651076"/>

<concentration name="ACACB" object_ID="953" value="41.177947"/>

<concentration name="MAOB" object_ID="3779" value="30.146111"/>

<concentration name="NPR1" object_ID="4278" value="31.122801"/>

<concentration name="ATP1A1" object_ID="13408" value="1195.998612"/>

<concentration name="NOS2" object_ID="4264" value="23.529520"/>

<concentration name="PAPOLG" object_ID="18784" value="117.661620"/>

<concentration name="IL4I1" object_ID="7937" value="14.828492"/>

<concentration name="GMPPB" object_ID="2929" value="48.765874"/>

<concentration name="ATP1A4" object_ID="13727" value="12.423502"/>

<concentration name="TREH" object_ID="6099" value="11.728934"/>

<concentration name="PIP5K1C" object_ID="4665" value="77.902623"/>

<concentration name="BCKDHB" object_ID="1444" value="144.397304"/>

<concentration name="TUFM" object_ID="17608" value="413.860285"/>

<concentration name="RIMKLB" object_ID="18390" value="68.261490"/>

<concentration name="RIMKLA" object_ID="18259" value="15.074100"/>

<concentration name="NMNAT1" object_ID="4253" value="83.032256"/>

<concentration name="CYP4A11" object_ID="2165" value="18.864474"/>

<concentration name="UPRT" object_ID="6259" value="246.868679"/>

<concentration name="GLUD1" object_ID="2922" value="911.184882"/>

<concentration name="CHDH" object_ID="1872" value="15.486079"/>

<concentration name="GMDS" object_ID="2926" value="170.390371"/>

<concentration name="ADCY9" object_ID="1041" value="95.803071"/>

<concentration name="GBE1" object_ID="2852" value="2296.148128"/>

<concentration name="LDHC" object_ID="3644" value="21.362373"/>

<concentration name="ACACA" object_ID="952" value="731.391475"/>

<concentration name="ATP6V0D2" object_ID="1357" value="16.621746"/>

<concentration name="LPIN1" object_ID="17359" value="493.976979"/>

<concentration name="ALDOB" object_ID="1137" value="12.613528"/>

<concentration name="GAPDHS" object_ID="2833" value="14.747515"/>

<concentration name="PDE4B" object_ID="4513" value="352.798349"/>

<concentration name="ATP2B3" object_ID="1332" value="13.839309"/>

<concentration name="NANP" object_ID="4103" value="273.367893"/>

<concentration name="CDA" object_ID="1762" value="18.069692"/>

<concentration name="PDE10A" object_ID="4506" value="14.625358"/>

<concentration name="HAO2" object_ID="3105" value="18.911606"/>

<concentration name="PAPD4" object_ID="6595" value="278.493530"/>

<concentration name="MRI1" object_ID="7247" value="1033.985329"/>

<concentration name="ADCY4" object_ID="1036" value="30.605096"/>

<concentration name="ENTPD1" object_ID="2481" value="14.212614"/>

<concentration name="AK7" object_ID="1096" value="13.270033"/>

<concentration name="LDHD" object_ID="3645" value="14.450024"/>

<concentration name="GAA" object_ID="2787" value="67.639777"/>

<concentration name="ACSM2A" object_ID="17944" value="1"/>

<concentration name="SMS" object_ID="5637" value="1"/>

<concentration name="DGKG" object_ID="2255" value="26.402452"/>

<concentration name="AHCYL2" object_ID="1083" value="46.109752"/>

<concentration name="BDH2" object_ID="1460" value="32.505231"/>

<concentration name="CDIPT" object_ID="1796" value="3550.976260"/>

<concentration name="PLCB2" object_ID="4705" value="27.109000"/>

<concentration name="AK5" object_ID="1095" value="32.091070"/>

<concentration name="AMY2A" object_ID="1177" value="1"/>

<concentration name="AMT" object_ID="1176" value="60.656906"/>

<concentration name="ADH1B" object_ID="1043" value="13.759918"/>

<concentration name="ATP11C" object_ID="1324" value="49.856165"/>

<concentration name="AGPAT4" object_ID="1076" value="67.630401"/>

<concentration name="PYGB" object_ID="5108" value="465.262145"/>

<concentration name="HIBCH" object_ID="3159" value="589.562111"/>

<concentration name="ZCCHC11" object_ID="14966" value="301.873436"/>

<concentration name="FASN" object_ID="2619" value="14.737297"/>

<concentration name="PLCD3" object_ID="4709" value="64.893407"/>

<concentration name="POLM" object_ID="4778" value="27.569477"/>

<concentration name="RBKS" object_ID="5224" value="191.991682"/>

<concentration name="ADCY10" object_ID="1033" value="17.431200"/>

<concentration name="HMGCS2" object_ID="3189" value="14.252074"/>

<concentration name="NT5C2" object_ID="4331" value="184.733282"/>

<concentration name="ATP6V1D" object_ID="1364" value="2038.087110"/>

<concentration name="POLL" object_ID="4777" value="59.664463"/>

<concentration name="TK2" object_ID="5972" value="45.940670"/>

<concentration name="AMY2B" object_ID="1178" value="376.785595"/>

<concentration name="RENBP" object_ID="16820" value="22.935334"/>

<concentration name="OXSM" object_ID="4413" value="756.659281"/>

<concentration name="OLAH" object_ID="4387" value="12.693341"/>

<concentration name="DGAT1" object_ID="2245" value="133.824644"/>

<concentration name="ATP2C2" object_ID="12039" value="17.336010"/>

<concentration name="DPYD" object_ID="2332" value="310.489356"/>

<concentration name="ACADM" object_ID="957" value="2586.884632"/>

<concentration name="GAMT" object_ID="2829" value="85.829400"/>

<concentration name="PNPLA3" object_ID="4756" value="104.524693"/>

<concentration name="ATP8B4" object_ID="10671" value="11.802332"/>

<concentration name="ME3" object_ID="3881" value="29.082916"/>

<concentration name="ALDH9A1" object_ID="1135" value="2902.338348"/>

<concentration name="OGDH" object_ID="4382" value="74.465486"/>

<concentration name="QPRT" object_ID="5115" value="33.556027"/>

<concentration name="EEF1A1" object_ID="2411" value="371.495262"/>

<concentration name="IDH2" object_ID="3291" value="537.902170"/>

<concentration name="IDH3G" object_ID="3294" value="584.921821"/>

<concentration name="G6PC2" object_ID="2785" value="27.116517"/>

<concentration name="AADAC" object_ID="898" value="12.949280"/>

<concentration name="ATP5F1" object_ID="1340" value="7940.422180"/>

<concentration name="ENTPD8" object_ID="2487" value="1"/>

<concentration name="GPAT2" object_ID="52540" value="1"/>

<concentration name="HK1" object_ID="3175" value="2437.184914"/>

<concentration name="SDHC" object_ID="5447" value="170.095363"/>

<concentration name="PNLIPRP2" object_ID="11523" value="11.954650"/>

<concentration name="TOP3B" object_ID="15906" value="33.939655"/>

<concentration name="EHHADH" object_ID="2430" value="25.639567"/>

<concentration name="HYI" object_ID="3277" value="91.095002"/>

<concentration name="SDSL" object_ID="13460" value="69.787657"/>

<concentration name="PI4K2B" object_ID="4613" value="399.790395"/>

<concentration name="ATP11A" object_ID="1323" value="19.164972"/>

<concentration name="PDE2A" object_ID="4510" value="21.359412"/>

<concentration name="BPGM" object_ID="1495" value="310.295723"/>

<concentration name="PGM3" object_ID="4581" value="270.315420"/>

<concentration name="PFKFB4" object_ID="4559" value="193.622124"/>

<concentration name="PIP5K1A" object_ID="4663" value="324.438226"/>

<concentration name="ENTPD6" object_ID="2485" value="317.079325"/>

<concentration name="POLH" object_ID="4774" value="108.368376"/>

<concentration name="ACAT2" object_ID="964" value="2805.417943"/>

<concentration name="ATP6AP1" object_ID="1350" value="959.208079"/>

<concentration name="ALDH7A1" object_ID="1134" value="33.946713"/>

<concentration name="POLE3" object_ID="4770" value="1719.770098"/>

<concentration name="GYS1" object_ID="16618" value="202.208523"/>

<concentration name="PDHB" object_ID="630" value="2717.378848"/>

<concentration name="ATP8A1" object_ID="7865" value="13.257161"/>

<concentration name="LCT" object_ID="3639" value="18.864474"/>

<concentration name="IDH3B" object_ID="3293" value="1309.681235"/>

<concentration name="AMPD2" object_ID="1174" value="384.755996"/>

<concentration name="PLCD1" object_ID="4708" value="131.899931"/>

<concentration name="ATP6V1C1" object_ID="1362" value="310.145203"/>

<concentration name="ATP2A1" object_ID="15059" value="18.167651"/>

<concentration name="UGT2A1" object_ID="6235" value="1"/>

<concentration name="CTH" object_ID="2097" value="332.497573"/>

<concentration name="GATM" object_ID="2848" value="13.635541"/>

<concentration name="PI4K2A" object_ID="4612" value="142.814633"/>

<concentration name="ASNS" object_ID="1306" value="4383.902509"/>

<concentration name="ATP8A2" object_ID="1374" value="17.213875"/>

<concentration name="PDHA2" object_ID="4532" value="18.228197"/>

<concentration name="UCK1" object_ID="6215" value="188.667223"/>

<concentration name="PLCB3" object_ID="4706" value="64.480901"/>

<concentration name="PAPOLA" object_ID="18369" value="1588.005287"/>

<concentration name="SDHD" object_ID="5448" value="4205.320379"/>

<concentration name="DLST" object_ID="2298" value="624.474997"/>

<concentration name="ATP2A2" object_ID="8402" value="273.064887"/>

<concentration name="PDE1B" object_ID="7062" value="54.244313"/>

<concentration name="POLR3B" object_ID="4798" value="652.801246"/>

<concentration name="HSD17B4" object_ID="3244" value="3048.745483"/>

<concentration name="PRPS2" object_ID="4993" value="1467.352730"/>

<concentration name="GOT1" object_ID="2974" value="963.339158"/>

<concentration name="PCK2" object_ID="4486" value="553.830233"/>

<concentration name="PDE8B" object_ID="4524" value="17.774043"/>

<concentration name="ENO1" object_ID="2470" value="3023.491903"/>

<concentration name="POLB" object_ID="4762" value="1188.560808"/>

<concentration name="ATP5I" object_ID="1345" value="4144.550711"/>

<concentration name="NOS3" object_ID="4265" value="17.656164"/>

<concentration name="ATP6V1B1" object_ID="1360" value="19.060314"/>

<concentration name="MGAM" object_ID="3908" value="1"/>

<concentration name="PYGM" object_ID="5110" value="28.656689"/>

<concentration name="PDE9A" object_ID="4525" value="12.193159"/>

<concentration name="PI4KB" object_ID="4615" value="173.476929"/>

<concentration name="GPI" object_ID="2986" value="936.022890"/>

<concentration name="ACSS1" object_ID="990" value="46.510998"/>

<concentration name="ATP6V1F" object_ID="1367" value="1586.904947"/>

<concentration name="BHMT" object_ID="1470" value="30.820108"/>

<concentration name="AGXT2L2" object_ID="8663" value="126.544413"/>

<concentration name="GMPPA" object_ID="2928" value="221.076214"/>

<concentration name="APRT" object_ID="1238" value="622.918669"/>

<concentration name="C9orf95" object_ID="1619" value="117.059645"/>

<concentration name="MYBBP1A" object_ID="17406" value="135.749211"/>

<concentration name="PYGL" object_ID="5109" value="771.648493"/>

<concentration name="ADK" object_ID="1050" value="1451.169174"/>

<concentration name="KHK" object_ID="3525" value="142.359997"/>

<concentration name="TXNRD2" object_ID="6176" value="81.577534"/>

<concentration name="DGKZ" object_ID="2260" value="798.085775"/>

<concentration name="ADCY2" object_ID="1034" value="22.181730"/>

</Cell>

…

…

</CancerCells>
