# Supplementary material for: Computational modeling of methionine cycle-based metabolism and DNA methylation and the implications for anti-cancer drug response prediction
Source: Oncotarget. 2018 Feb 21;9(32):22546–58. doi: 10.18632/oncotarget.24547 (PMC5989406; doi:10.18632/oncotarget.24547)
Supplement: Supplementary file 4 [file oncotarget-09-22546-s004.doc]

Supplementary Information 3: Kinetic Laws from AutoAnalyse

| **Reaction** | **Kinetic rate law** | **Optional** | **Constants** |
| --- | --- | --- | --- |
| Acetylation |  |  |  |
| activation |  |  |  |
| complex |  |  |  |
| decay |  |  |  |
| dephosphorylation |  |  |  |
| dimerization |  |  |  |
| metabolism |  |  |  |
| methylation |  |  |  |
| mirna binding |  |  |  |
| oxidization |  |  |  |
| phosphorylation |  |  |  |
| transcription |  |  |  |
| translation |  |  |  |
| translocation |  |  |  |
| ubiquitnation |  |  |  |
| default / unkown |  |  |  |
